# From Genetic Determinism to Epigenetic Regulation: Paradigm Shifts in the Understanding of Neurodevelopmental Disorders

**DOI:** 10.3390/cimb48020163

**Published:** 2026-02-02

**Authors:** Ernesto Burgio, Annamaria Porru, Chiara Pettini, Ilaria Vaglini, Angelo Gemignani, Marco Pettini, Federica Fratini, Daniela Lucangeli

**Affiliations:** 1 European Cancer and Environment Research Institute, 1000 Bruxelles, Belgium; erburg@gmail.com; 2 Department of Developmental Psychology and Socialisation, University of Padova, 35131 Padova, Italy; annamaria.porru@unipd.it (A.P.); chiara_pettini@libero.it (C.P.); ila.vaglini@gmail.com (I.V.);; 3 Department of Surgical, Medical, and Molecular Pathology and Critical Care, University of Pisa, via Savi 10, 56126 Pisa, Italy; angelo.gemignani@unipi.it; 4 Center for Theoretical Physics, Aix-Marseille University, CNRS, 13288 Marseille, France; marco.pettini@univ-amu.fr; 5 Quantum Biology Laboratory, Howard University, Washington, DC 20060, USA; 6 Neuroscience Department, Italian National Health Institute, 00161 Rome, Italy; 7 Mind4Children, University of Padova, 35131 Padova, Italy

**Keywords:** epigenetics, neurodevelopmental disorders, epigenetic inheritance, systems biology, neurodevelopmental risk factors, neurodevelopmental salutogenic factors

## Abstract

Over the past two decades, advances in the understanding of epigenetic mechanisms—driven by the rapid expansion of omics technologies—have catalyzed a major paradigm shift in biology: from the genetic determinism and linear causality of the Central Dogma toward the dynamic, networked complexity of systems biology and multilevel regulation. This reconceptualization extends to inheritance itself, highlighting the crucial role of the epigenome as a molecular interface between the genome and the exposome—the cumulative set of internal and external environmental influences experienced across the lifespan. Within this evolving framework, neurodevelopmental disorders exemplify the deep entanglement between genetic predisposition, environmental exposure, and epigenetic modulation. Their increasing global prevalence and frequent comorbidities underscore the need for an integrated etiological understanding that transcends reductionist models. This review tries to synthesize current evidence on the shared molecular and systemic mechanisms underlying neurodevelopmental spectrum disorders and examines how environmental and epigenetic factors jointly shape neurodevelopmental trajectories across generations. Finally, it discusses the broader implications of this paradigm shift for early diagnosis, prevention, and public health policies aimed at fostering healthy brain development in future generations.

## 1. Introduction

Neurodevelopmental disorders (NDDs), including autism spectrum disorders (ASDs), Attention-Deficit/Hyperactivity Disorder (ADHD) and certain neuropsychiatric conditions like schizophrenia (SZ), have long been considered primarily genetic in origin. This perspective has been supported by extensive genetic studies that highlight heritable components underlying these disorders. However, a striking and persistent rise in their prevalence over recent decades and the high rates of comorbidity across several psychiatric disorders suggest a paradigm shift from genetics to epigenetics, from linearity to complexity, from nodes to edges. In the scientific understanding of neurodevelopmental conditions, in fact, there is strong evidence that genetic factors cannot be the unique determinants; epigenetic mechanisms rather serve as crucial mediators in a complex interplay of genetic predisposition, neurobiological pathways, and environmental influences [1]. A significant development in understanding these interconnections is the discovery of shared neural basis and genetic architecture, or pleiotropy. Studies indicate that several psychiatric and neurodevelopmental disorders, including ASD, ADHD, SZ, Bipolar Disorder, other personality disorders, Eating Disorders, and Major Depressive Disorders (MDD), may share common neurobiological pathways, specific temporal windows of susceptibility, and vulnerable brain regions [2,3,4,5].

This evidence strongly suggests that these conditions cannot be seen as entirely separate entities genetically determined. Rather, different phenotypic expressions could arise from variations in a common set of fundamental brain development and function pathways, emerging from a dynamic interplay between individual genetic predisposition and the environmental factors faced during development, with epigenetic modifications serving as critical mediators of this interaction. This perspective could fundamentally shift the understanding of comorbidity from a mere chance of co-occurrence to a manifestation of shared neurodevelopmental vulnerabilities. Recent studies have increasingly pointed to the role of epigenetic mechanisms in shaping neurodevelopmental outcomes [6], particularly in response to environmental stressors. Further, despite a several-year-long-standing controversial debate on the transgenerational epigenetic inheritance in humans [7,8], a very recent paper provides some compelling evidence that trauma and adverse experiences may leave stable epigenetic marks across generations [9]. This research highlights how environmental factors—especially extreme stressors such as war and migration—can influence gene expression in ways that extend beyond an individual’s direct experience, potentially predisposing future generations to neurodevelopmental and psychiatric disorders.

The present article, hovering between a review and a perspective article, examines—without pretending to be exhaustive—the scientific evidence in support of a neuro–bio–psycho–social model. In our opinion, such a model may, on the one hand, account for the epidemiological increase, especially among children and adolescents, and the comorbidity of various NDDs; and on the other hand, critically support the need not only for personalized therapeutic and educational approaches for symptomatic people, but above all, for socio-educational-health policy interventions on environmental factors, particularly for preventing early life adversities and promoting healthy and safe human development. In light of the emerging evidence on risk factors and epigenetic modifications, we propose a reassessment of the genomic framework, which may offer a more nuanced understanding of these complex disorders, helping to bridge the gap between genetic predisposition and dynamic environmental influences.

**Figure 1:**
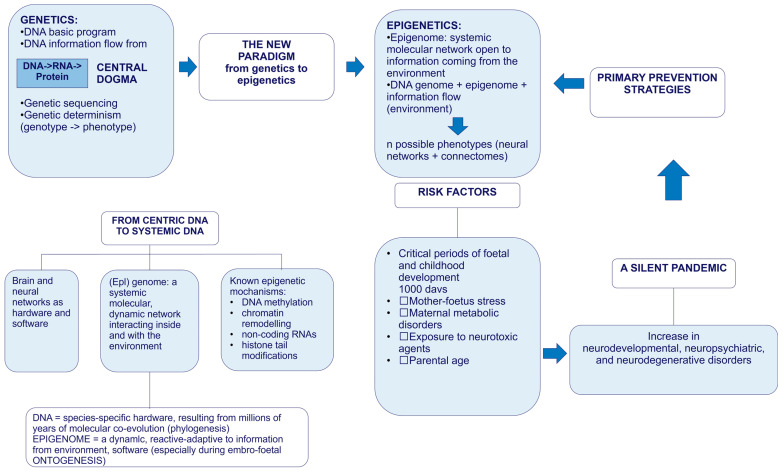
Explanatory chart.

Figure 1 provides a synoptic explanatory chart of the content of the present paper.

## 2. Towards a New Paradigm: From Linear Genetics to Systems Biology and Complex Genomics (Epigenetics, Metagenomics, and Hologenomics)

**Figure 2:**
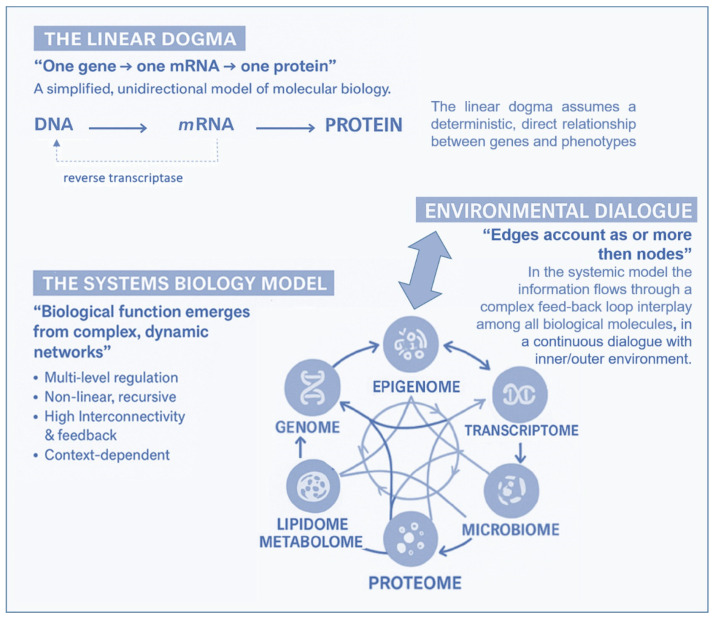
According to the “Central Dogma” of molecular biology, information flows unidirectionally from DNA, the site of the basic genetic program, through transcription into mRNA and translation into proteins, to the individual phenotype. The pattern is linear and leaves no room for any reversible information flows, less than reverse-transcription. In the systemic model, instead, the information flows through a complex feedback loop interplay among all biological molecules, in a continuous dialog with the inner/outer environment.

Over the past two decades, advances in molecular biology, particularly in -omics disciplines (genomics, transcriptomics, proteomics, metabolomics, and lipidomics), epigenetics, and metagenomics (the study of entire genomes within an environmental sample, offering insights into microbial communities), have expanded exponentially. The relevant knowledge acquired has led to a revision of the linearity of the “central dogma of biology” in favor of a new systemic biological model. The classical gene-centered model has been refined by a context-dependent gene expression and the emergence of a phenotype as a complex, dynamic, highly interconnected, and interactive network of informative molecules within their environment. In this new network model, the edges account for more than the nodes, and, of particular importance, it is its most adaptable component, the epigenome, which some molecular biologists, pioneers in the field of environmental epigenetics, referred to as the “DNA software” [10]. In the traditional framework (Figure 2)—describing a linear, one-way flow of genetic information from DNA to messenger RNA (mRNA) to proteins, which then form the structural and functional basis of the phenotype—DNA is regarded as the fundamental genetic program, with phenotypic variations, both physiological and pathological, attributed primarily to changes in the DNA sequence. The reverse information flow is not possible, except in rare cases, such as reverse transcription. For nearly half a century, DNA was conceptualized as a static repository of genetic information, the result of millions of years of molecular evolution, and largely unchanged over time. According to this view, the human genome was considered 99% identical among individuals of the same species (*Homo sapiens sapiens*) and highly similar to closely related species. For instance, the genomes of chimpanzees and humans share approximately 98% of their coding sequences, which, under the dominant model, were regarded as the most functionally significant components. Specifically, DNA was thought to contain a fixed blueprint for constructing an organism, with the genotype acting as an unalterable program that determined phenotype. It was widely believed that the coding regions of DNA were the most crucial and were therefore highly conserved throughout evolution. As a result, studying the DNA sequence and its variations—whether neutral (polymorphisms) or potentially pathogenic (mutations)—was expected to be sufficient to explain both physiological traits and disease development. This philosophy formed the foundation of one of the most ambitious biomedical research projects of recent decades: The Human Genome Project; the sequencing of the entire human genome (and that of many other species) should have led to the knowledge of every genetic and/or protein alteration. However, the findings of this extensive research project diverged significantly from expectations. The first evidence was the partial failure of the genome-based prediction of transcriptome and proteome (in terms of mRNA, lncRNA, and post-translational-modifications of proteins), revealing a much higher complexity in the regulation of gene expression (Figure 2).

**Figure 3:**
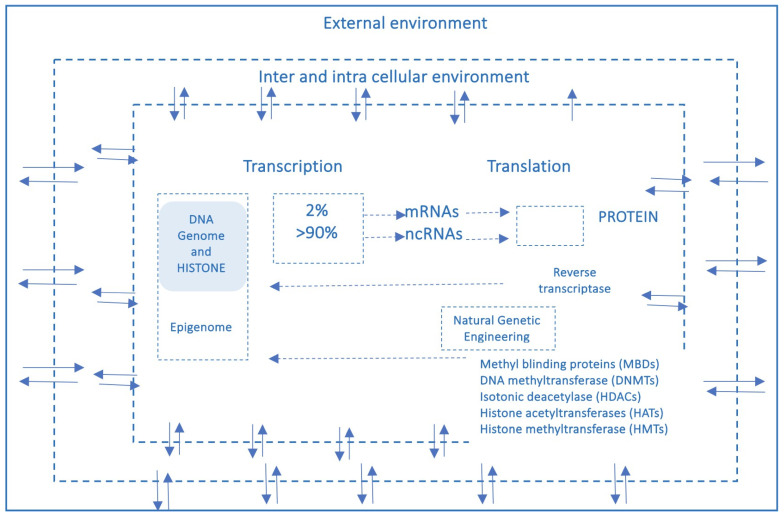
This figure provides a systemic and dynamic view of the genome, pointing out how continuous information exchange (arrows) between the epigenome and the environment would be. Unlike the linear pattern (Central Dogma), the main direction of information flow goes from the environment to the system.

Moreover, the rapid global increase in chronic diseases [11] can hardly be attributed solely to an improved public health and long-lived population, and much less to a sudden surge in random genetic mutations or polymorphisms. Growing evidence suggests that these conditions result from interactions between genetic and environmental factors and may originate from early-life epigenetic alterations driven by the dynamic interplay with the exposome across the lifespan [12]. Unlike genetic mutations, epigenetic modifications are potentially adaptive and responsive to environmental cues. This means that the fetus also adjusts its epigenetic programming in response to environmental signals received through the mother—a process known as fetal programming. Growing evidence suggests that early experiences may lead to changes in the epigenome, influencing metabolic and physiological pathways, possibly changing an individual’s phenotypic development and thus having a critical effect on their lifelong health [13]. It has been reported that crucial developmental mechanisms can be disrupted by various factors, including premature birth, maternal stress, exposure to environmental pollutants (found in the umbilical cord and placenta), and maternal inflammatory or metabolic disorders [14,15]. Both the data obtained from the Human Genome Project (2000) and the growing body of research in molecular epigenetics support the proposed paradigm shift, which moves away from the traditional, static view of linear genetics toward a systemic, complex, interconnected and—most importantly—open genome model, highly responsive to environmental influences, both within the body’s microenvironment and the external world. A network model which strongly indicates that in living organisms, the dynamics of edge connections are more determinant than the presence/role of nodes. This paradigm shift is at the base of another enormous and ambitious project: The Human Exposome Project, 2020, from the European Union. This is the largest network of research programs aiming to address how environmental exposure, such as diet, lifestyle, occupational, and other environmental factors, has an impact on human health (https://www.humanexposome.eu, accessed on 1 January 2026). The new genomic model conceptualizes DNA as a stable database—akin to hardware—that is largely unchanged over time. However, other chromatin components, such as (i) histone protein terminal “tails”; (ii) key enzymes essential for dynamic genetic expression and epigenetic reprogramming; (iii) noncoding RNAs, which form a complex, open molecular system that, responding to environmental signals, define the chromatin structure and accessibility for gene expression. These interactions drive morpho-functional modifications that shape cell fate and behavior, ultimately defining the phenotype from early embryonic development onward. This new paradigm, which emerged from molecular biology research—particularly during the ENCODE project [16]—deepens and redefines the linear, preordained view of DNA-to-protein information flow. Indeed, it suggests a systemic model, where information originates from the environment (particularly from the body’s microenvironment) and is transmitted through intracellular and intercellular signaling networks. These signals influence the epigenetic regulatory system, which, in turn, interacts with DNA. In this model, continuous epigenetic modifications—particularly three-dimensional changes in chromatin architecture, such as histone tail modifications and DNA methylation—are not merely passive or random but rather reactive and potentially adaptive. Figure 3 presents a systemic and dynamic view of the genome, highlighting the continuous bidirectional exchange of information between the epigenome and the environment. Extending the linear model of the Central Dogma, where information flows unidirectionally from DNA to RNA to proteins, the primary direction of information flow in the systemic model originates from the environment and influences the entire genomic system. In support of this model, we know that only 2% of DNA is transcribed into messenger RNA (mRNA) and subsequently translated into proteins, which form the structural and functional basis of the phenotype. In contrast, non-coding RNAs (ncRNAs)—which make up more than 90% of all transcripts—appear to be the key molecular regulators of the entire system. Proteins, both enzymatic and structural, ultimately determine phenotypic expression. However, in the systemic model, proteins are not the direct and exclusive products of genes. Instead, they are the result of highly sophisticated and complex molecular processes, which are triggered by environmental inputs and modulated by the epigenome. This concept aligns with the Natural Genetic Engineering theory proposed by Shapiro [17]. Interestingly, reverse transcriptase—an enzyme used by retroviruses to reverse transcribe RNA into DNA and integrate it into the host genome—appears to play a similar role in the genomic system of higher organisms [18,19]. The epigenome and microbiome [20] (the unique genome of an individual’s microbial ecosystem) are the primary modulators of the entire genomic system. Through their interactions, environmental signals dynamically shape and regulate the genome, influencing gene expression and phenotype. Based on these new understandings, it can be easily postulated that chronic, degenerative, inflammatory, neuropsychiatric, and neoplastic diseases ultimately emerge from a prolonged, reactive, and adaptive process—one that is initially potentially protective but becomes progressively disrupted, particularly in the first 1000 days, by constant environmental changes. Chemical (molecular) factors, like pesticides or air pollution or microplastics/nanoplastics; physical influences, such as electromagnetic fields and ionizing radiations or malnutrition; or internal environments, meaning not only individual genomic predisposition but also the psychophysical wellbeing, such as stress, violence, trauma, addictions, are all factors that influence the epigenome and microbiome, both inter-generationally [21] and potentially also trans-generationally [9].

## 3. The Dynamic Interplay Between Nature and Nurture: An Epigenetic Shift

Many researchers have long debated the respective roles of environmental influences (Nurture) and genetic inheritance (Nature) in disease development and phenotype transformation [22]. In recent years, however, the Nature vs. Nurture debate has undergone a fundamental advance thanks to the understanding of epigenetic mechanisms through which environmental factors elicit potentially lifelong biological changes, influencing gene expression without altering their underlying DNA sequence. The concept of gene-environment–epigenome dynamic interplay offered a sophisticated and comprehensive framework that effectively bridges the long-standing Nature–Nurture debate between an individual’s genetic predispositions and their environmental exposures throughout life [23]. A key concept arising from this interaction model is “biologically embedding”, i.e., encoding, preserving, and transmitting. Early changes in DNA methylation, for instance, are proposed to dictate how an organism responds to stressors later in life, effectively enabling adverse childhood experiences to become biologically “embedded” by leaving genetic traces with life-long consequences [24,25]. This dynamic interplay model could represent a profound conceptual leap, effectively resolving the Nature–Nurture dichotomy by suggesting that genetic predispositions are not fixed destinies, but rather potential outcomes continuously shaped by environmental inputs via epigenetic modifications. From this perspective, it is becoming increasingly irrelevant to ask whether external environmental information or internal genetic programming plays a greater role in shaping an individual’s health and development. From this emerging framework, several key insights can be drawn:

1. The genome consists of two main components: a relatively stable part—the funda- mental DNA molecule, which serves as the molecular memory of a species and changes very slowly under natural conditions—and a more complex, dynamic part that interacts with the environment and is constantly evolving. This dynamic component corresponds to what we call the epigenome, the “software” that regulates genetic operation [26,27].
2. Everything we eat, breathe, listen to, feel, think, and suffer [28] influences the structure of the epigenetic software. Any environmental input (Nurture) can activate or deactivate a wide range of biochemical and intercellular circuits [15,29,30].
3. When scientists examine the genomes of two monozygotic twins at a very young age, they find them to be nearly identical (Nature). However, if they analyze the genomes of the same twins years later—after each has undergone different life experiences that gradually “mark” the epigenome and reposition chromatin—they will observe significant structural changes induced by life experience and environmental factors (Nurture) [31,32,33].
4. Epigenetic transformations are largely passed down from one cell generation to the next, facilitating and stabilizing the gradual morpho-functional differentiation of cells in various tissues. Increasing evidence shows that some epigenetic marks in gametes are preserved and transmitted from one human generation to another, potentially leading to the inheritance of damage and even predisposing individuals to neoplastic diseases [34,35,36,37].
5. Major epigenetic transformations primarily affect less differentiated cells, which have a more flexible genomic structure. These include pluripotent cells in the early stages of embryonic and fetal development, as well as stem cells in various tissues. The epigenetic marks on germ cells (gametes) can have an impact on the health of future grandchildren [38]. In this light, if we truly consider the environment as a continuous flow of stimuli and molecular information, we could hypothesize that every cell—and, by extension, every organism—is constantly compelled to change in order to adapt. Since this would represent its fundamental purpose, it could not only acquire and process, but also convert this incoming information into new, stored biological data. Initially, these changes could occur at the epigenetic level, but over time, probably with the persistence of stimuli, they could become embedded at the genetic level.

## 4. Epigenetic Intergenerational and Transgenerational Inheritance

**Figure 4:**
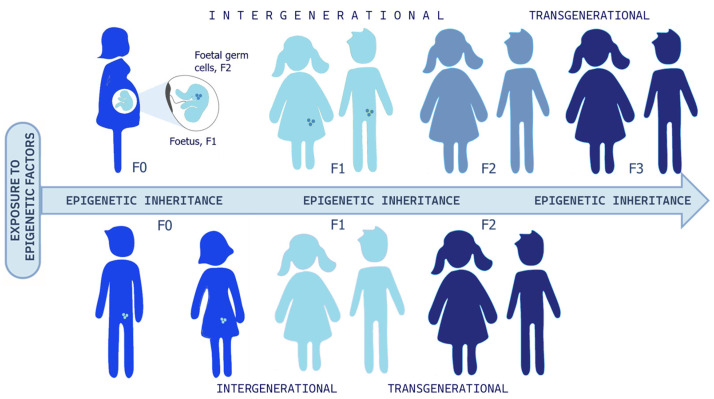
Epigenetic inheritance. If a pregnant woman (F0) is exposed to epigenetic factors, the fetus (F1) and its germ cells (represented as little circles in F1, and as developed human being in F2) are also directly exposed, and this could result in intergenerational transmission till the F2 offspring. In this case, the third generation (F3) is the first one that could represent transgenerational epigenetic inheritance. Otherwise, if a man or woman (F0) and their germ cells (F1) are exposed to environmental stressors, this could result in intergenerational transmission until the F1 generation, and the F2 offspring could signify transgenerational epigenetic inheritance. Readapted from Ref. [41]. A significant portion of human studies has focused on the intergenerational transmission of stress-related effects from parents to offspring [40]. For example, Yehuda et al. found that pregnant women who developed Post-Traumatic Stress Disorder (PTSD) following a traumatic event—such as the evacuation from the World Trade Center on 11 September 2001—gave birth to children with cortisol levels mirroring those of their mothers [51,52]. This effect was observed when the traumatic exposure occurred during the third trimester of pregnancy, but not during the second trimester. These findings support the idea that certain stages of pregnancy are particularly sensitive to in utero transmission of biological vulnerability factors from mother to fetus. This underscores the importance of the first 1000 days of a child’s life in shaping long-term health outcomes [53,54].

Research has shown that exposure to chemical, physical, or psychological stressors during pregnancy can lead to epigenetic modifications. These changes may jeopardize fetal development and increase the risk of diseases not only in childhood but also in adulthood. Further, when epigenetic modifications affect gametes or germ cells, they can be passed on to future generations [39,40,41] and Figure 4. In other words, living in an unhealthy environment during pregnancy—during critical “windows of exposure”—can negatively impact the mother, the fetus, and the fetus’s germ cells, resulting in an intergenerational epigenetic inheritance. In this way, the risk of disease can extend up to three successive generations, or even more. There is an open debate on the possibility of transgenerational epigenetic inheritance that, if true, would change our understanding of evolutionary mechanisms significantly. The challenge lies in distinguishing the existence of epigenetic memory that instructs de novo epimutation of germlines, meaning a real transmission of altered epigenetic marks, or whether these marks are first erased and then re-established during development, for the persistence of the exposure to environmental stressors. Transgenerational inheritance has been shown in several animal models, and the recent paper of Mulligan would support transgenerational inheritance (third generation) in one human case [9,35,42,43,44]. Nevertheless, this intriguing topic is still controversial, and drawing parallels between animal models and humans is arduous. In fact, while longitudinal studies should be funded to follow three or more generations and carefully analyze confounding factors and social transmission pathways, it cannot be ignored that in the real lives of humans, apart from sporadic traumatic episodes, it is very rare for the ecological, social, and cultural environment to differ significantly from one generation to the next. On the other hand, if confirmed in animal models, we should ask for the adaptive evolutionary significance and why this mechanism should not be conserved in such a more complex and maybe younger species, such as *H. Sapiens*. In our opinion, besides the huge importance of understanding evolutionary mechanisms, the emerging evidence of environmental influence on healthy development is enough to argue for environmental justice [45] and salutogenic approaches, like enriched environments, as well as cultural and psychosocial interventions, having shown the potential to mitigate trauma’s impacts within and across generations [36,40,46,47,48,49,50].

## 5. A Historical/Epistemological Digression

It is not out of place to make a historical digression because the modern findings on epigenetics resonate strikingly with Aristotelian insights into the continuity of life. Aristotle conceptualizes life as a principle that perpetuates itself through generations, where each organism carries within itself the potential for the continuation of the species [55]. In *De Respiratione*, the ancient philosopher argues the importance of the external environment for the maintenance of life and conceiving breathing as a vital element that connects the organism to the surrounding world [56]. Finally, in *De Vita et Morte*, he describes death as a natural process intrinsic to life itself, where the initial conditions determine the duration and quality of existence [57]. These conceptions seem to find now an extraordinary echo in the modern understanding of how early environmental factors can “program” the biological course of the individual, influencing not only his life but also that of future generations. We find greatly interesting the reflection on the convergence between epigenetic inheritance and Aristotelian philosophy, uniting in a temporal continuum the individual experience and the collective experience of the species. It suggests, behind any phenomenon, a structural and dynamic complexity that transcends individuality to encompass a multigenerational time horizon and the evolution of species. From an epistemological standpoint, these findings motivate a life-course framework in which early-life conditions can become biologically embedded and interact with genetic susceptibility. Practically, this perspective encourages study designs that model developmental timing and cumulative exposures, and that explicitly test plausible biological mediators (e.g., inflammatory, endocrine, and epigenetic pathways) rather than treating genes and environment as separable, single-cause explanations. In this paradigm, individual genetic determinism converges with social epigenetic responsibility towards the environment, which takes on an ethical dimension that goes beyond the present, extending to a chain of causality—about the importance of initial conditions for later trajectories of health and disease—that Aristotle had intuited and that modern science seems to be confirming through epigenetic mechanisms [58].

## 6. Epigenetic Signatures in the Spectrum of Neurodevelopmental Disorders

Neurodevelopmental disorders are defined as a group of conditions that manifest during the developmental period, leading to deficits that impair functioning. These impairments typically affect cognition, communication, behavior, and/or motor skills, and are understood to result from abnormal brain development [59].

A range of common NDDs includes ASD, ADHD, Intellectual Disability (ID), SZ, Specific Learning Disorders (SLDs), Communication Disorders, Developmental Coordination Disorder, and Neurodevelopmental Motor Disorders (including Tic Disorders and Tourette Syndrome). A notable characteristic of NDDs is their significant clinical heterogeneity and the frequent lack of precise diagnostic boundaries. Many symptoms overlap across different disorders; for example, impaired social cognition is a common feature observed in both ASD and SZ [60]. The “Neurodevelopmental Disorders” chapter in the DSM-5 reflects a growing scientific understanding of the shared neurobiological underpinnings, developmental trajectories, and the complex, often co-occurring, nature of these conditions, resulting in high rates of comorbidity observed across NDDs. This pervasive overlap strongly suggests shared underlying epigenetic mechanisms, genetic vulnerabilities, and environmental factors that contribute to multiple developmental challenges simultaneously [61].

Autism Spectrum Disorders (ASDs) refer to a group of neurodevelopmental conditions characterized by difficulties in social interaction and communication, along with repetitive behaviors and restricted interests. The clinical presentation of ASD is highly heterogeneous, primarily due to the significant variability in cognitive functioning. This can range from severe intellectual disability to normal or even above-average intelligence. The broad phenotypic variability, the continuous increase in suspected or diagnosed cases, and the overlap of symptoms with other neurodevelopmental disorders—such as ADHD and obsessive–compulsive disorders—suggest that these conditions are linked to synaptogenesis and neural network formation. Emerging evidence supports shared neurobiological mechanisms between NDDs and mood disorders, including genetic susceptibilities, neurotransmitter dysregulation, neuroinflammation, hypothalamic–pituitary–adrenal (HPA) axis dysfunction, and altered connectivity in brain regions critical for emotional regulation and executive function, such as the prefrontal cortex and amygdala [62]. This perspective raises the possibility that ASD and related conditions may be epigenetically driven rather than purely genetic, and thus, potentially modifiable or to some extent reversible. Extensive epidemiological studies, particularly those involving twins, along with the frequent recurrence of ASD in family histories, have long led to the assumption that ASD—like SZ—is primarily a genetic disorder. However, it is important to reconsider what is actually meant by the term “genetic disorder”. This term is traditionally used to describe single-gene diseases and chromosomal abnormalities—such as trisomy 21 (Down syndrome)—which are typically inherited and passed from one generation to the next. In contrast, many chronic diseases—including obesity, type 2 diabetes in children, neurodevelopmental and neuropsychiatric disorders, neurodegenerative diseases, immunomediated conditions, and cancers—do not involve specific, heritable genetic mutations. Instead, their underlying genomic modifications are primarily epigenetic in nature, often originating as early as the embryonic or fetal stage. Even genetic variations associated with neurodevelopmental disorders and major depressive disorders follow this pattern. These variations are often due to main epigenetic signatures like DNA Methylation, Histone modification, lncRNAs, and copy number variations (CNVs) affecting genes crucial for brain development and neural network formation. Notably, these changes are reactive rather than hereditary and vary significantly from case to case. This variability is frequently observed in ASD and SZ [63]. Some of the main epigenetic signatures identified in the literature [1,64,65,66,67,68,69,70] in the most frequent NDDs are reported in Table 1.

These findings further support the hypothesis proposed by some researchers that neurodevelopmental disorders and major depressive disorders may exist along the same disease spectrum. The “spectrum” concept, now central to NDD classification, represents a fundamental philosophical shift in psychiatric nosology, moving away from a purely categorical “ill” or “healthy” dichotomy. Emphasizing the continuum of symptoms and functional impact encourages clinicians to think dimensionally. This is particularly evident in the ASD severity levels, which directly link symptom presentation to support needs [71]. This approach fosters a more person-centered model of care. It facilitates the development of highly individualized treatment plans and educational accommodations that are tailored to an individual’s specific functional profile and support requirements, rather than being dictated by a broad diagnostic label. It also promotes a greater appreciation for the diverse ways NDDs manifest and the varied strengths individuals with these conditions may possess, moving beyond a sole focus on deficits [72]. In clinical settings, autism spectrum disorders (ASDs) are diagnosed based on distinctive behavioral abnormalities, assessed using standardized diagnostic tools. However, this does not mean that diagnostic evaluation and treatment should be limited to this aspect alone. Potential disruptions during embryonic, fetal, or newborn development can affect more than just the nervous system. Therefore, we consider that a systemic approach is essential—one that considers the wide range of associated comorbidities. These may include gastrointestinal disturbances, psycho–neuro–immunological–endocrine disorders (particularly allergies), sleep disorders, and epilepsy [73]. In this direction, an effort is underway to realize suitable, digitalized, and user-friendly tools designed to systematically collect maternal and child environmental histories and inflammatory factors alongside symptom profiles for children with neurodevelopmental disorders, like the “NDD-ECHO”: Neurodevelopmental Disorders–Environmental and Clinical History Online [74].

## 7. Epidemiological Data: Genuine Increase or Improved Diagnosis?

A November 2006 article published in The Lancet, authored by a pediatrician and an epidemiologist from the Harvard School of Public Health, raised serious concerns about a “silent pandemic” of neurodevelopmental disorders spreading across the northern hemisphere amid widespread indifference [75,76]. This framing underscores concern about rising burden; however, observed increases in reported diagnoses reflect both ascertainment changes and, potentially, shifts in underlying risk. According to the DSM-5 (2013) criteria, NDDs represent a significant global health burden. Among people under 18 years old, the most recent prevalence [percentage of individuals meeting diagnostic criteria within a defined population] rates of NDDs reported 5–11% for ADHD; 0.70–3% for ASD; 3–10% for SLD [77]. Current evidence is supportive of a global increase in ASD prevalence over the past years, with 1.70 and 1.85% in U.S children aged 4 and 8 years, respectively, while prevalence in Europe ranged between 0.38 and 1.55% [78]. There remains considerable disagreement over the official epidemiological data provided by different countries, particularly regarding the actual rate of increase. Those who deny a significant rise in cases often attribute the apparent surge to refinements in diagnostic criteria. However, epidemiological data from multiple countries, particularly Anglo-Saxon nations, suggest a clear and alarming trend (Table 2). In the United States (US), the prevalence of ASD has risen from approximately 1 in 1500 children aged 8 years in the 1970s and 1980s to 1 in 150 in the early 2000s, then to 1 in 68 in 2014, 1 in 59 in 2016 [79]. More recent data from the Autism and Developmental Disabilities Monitoring (ADDM) Network of the US Centers for Disease Control (CDC) for the 2022 surveillance year (reflecting 8-year-old children born in 2014), indicates a further increase to 3.2% (or 1 in 31). A recent study on ASD trends in California from 1990 to 2018 analyzed diagnosis incidence by age 4 or 8 [that is, the percentage of new births developing ASD diagnosis within 4 and 8 years within the annual birth cohort], stratified by socioeconomic status (SES) and sociodemographic factors. The cumulative incidence showed an increased rate while the age of diagnoses decreased. As expected, a higher incidence was registered in lower SES with low education, while the age at first diagnosis showed the reverse trend [80]. Worldwide epidemiological surveys of ASD conducted in 37 countries reported a median prevalence of 0.77–1%. Many countries, particularly low and middle-income nations, lack sufficient data on ASD and exhibit a lower prevalence of 0.30%, possibly due to potential underdiagnoses or differences in healthcare access. High-income countries had a median prevalence of 0.86%. The global prevalence of ASD reaches the rate of 1.14 per 100 children among males [81,82,83]. Nevertheless, it is possible that prevalence estimates in ASD are underestimated for reasons lying in the complexity of the phenotypes and severity associated with the spectrum disorders; but also, in the absence of gold standard tools (maybe impossible) and a global surveillance network (possible), implying differences in the assessment of prevalence among regions and countries. International guidelines on ASD suggest that the diagnosis should be made by a multidisciplinary team of expert clinicians and caregivers, but this possibility is rare, and children can receive a misdiagnosis and inaccurate evaluations. It is also possible, in fact, that the prevalence could be overestimated, often in situations where ASD is associated with services and care that would not be otherwise granted for other disorders. Further, a real esteem for ASD prevalence is complicated by the high rates of comorbidities: ADHD (0.00–86.00%), anxiety (0.00–82.20%), depressive disorders (0.00–74.80%), epilepsy (2.80–77.50%), ID (0.00–91.70%), sleep disorders (2.08–72.50%), sight/hearing impairment/loss (0.00–14.90%/0.00–4.90%), and GI syndromes (0.00–67.80%) [61,81]. Prevalence of ASD has increased over time, while comorbidities bring additional heterogeneity to the clinical presentation, which further advocates for personalized approaches to treatment and support.

The rising trend has been attributed to a combination of factors, including (a) increased attention to regular pediatric screening in the first 24 months as recommended by the American Academy of Pediatrics in 2023; (b) the increasing awareness of clinicians, educators and parents more attentive and skilled to recognize early ASD signs; (c) improved diagnostic tools and changed diagnostic criteria; and (d) a genuine increase in ASD [80,81,82,83]. Biologically speaking, the main correlations with this dramatic increase are believed to be persistent maternal–fetal stress, chronic inflammation and/or infections, and, most notably, maternal–fetal exposure to thousands of synthetic and potentially neurotoxic compounds—such as pesticides, heavy metals, and endocrine disruptors—detected in the placenta, umbilical cord blood, and breast milk. Epidemiological and toxicological data collected in the years support the existence of a neurodevelopmental disorder pandemic, but also point to a similar trend in MDDs and neurodegenerative diseases, particularly Alzheimer’s disease. In 2014, again, Grandjean and Landrigan published a comprehensive and well-documented literature review in The Lancet Neurology, emphasizing the severe impact of environmental neurotoxins—especially heavy metals released into the air in major cities and pesticides introduced into the food chain [76].

It is equally important to highlight that, in recent decades, the increase has not been limited to neurodevelopmental disorders—which now affect one in six children in the U.S.—but extends to all major neuropsychiatric disorders, particularly major depression and, notably, youth depression. As a result, there is growing recognition of a continuum of neuropsychological disorders, leading some researchers to classify them collectively as early-onset neuronal development disorders. More specifically, neurodevelopmental disorders primarily affect the formative processes of the brain—such as neurogenesis, synaptogenesis, and early neuronal network programming—whereas major neuropsychiatric disorders predominantly impact later stages of brain development, particularly synaptic pruning and connection refinement [4]. Even the ongoing rise in neurodegenerative disorders, particularly Alzheimer’s disease, should be understood within this context [84]. Previously rare, Alzheimer’s disease has been steadily increasing across the northern hemisphere, with prevalence rates and future projections that are just as alarming as those for neurodevelopmental disorders. Notably, neither longer life expectancy nor improved diagnostic methods can fully account for this surge. Some authors have highlighted that, over the same period, there has been a similarly significant and ongoing increase in endocrine and metabolic diseases (such as childhood obesity and type 2 diabetes), inflammatory and immune-mediated disorders (including allergies and autoimmune diseases), and various cancers. This trend appears to signal a major epidemiological transition and, more importantly, could suggest a common early origin—stemming from impaired fetal tissue and organ development disrupted by the same previously mentioned factors. These disruptions are increasingly leading to earlier disease onset [15].

Several contributing factors have been identified, including the following:

- The rising prevalence of preterm births.
- Persistent maternal–fetal and early childhood stress.
- Chronic inflammation and subacute infections.
- Maternal autoimmune diseases, which result in the placental transfer of cytokines and antibodies that interfere with fetal neuronal network development.
- Exposure to hundreds of synthetic chemicals and byproducts of thermochemical reactions detected in the placenta, umbilical cord, and the wider environment.

This widespread exposure is also related to the following:

- Pervasive vehicle emissions.
- The extensive use of pesticides in agriculture.
- Household insecticide use.
- Plasticizers and other endocrine-disrupting chemicals interfere with the adult psycho–neuro–immune–endocrine system and act as pseudo-morphogens in embryos and fetuses. These mimetic molecules can alter cellular differentiation processes, ultimately affecting fetal tissue and organ programming (fetal programming [85]).

This issue falls within the scope of the Developmental Origins of Health and Disease (DOHaD) theory [86], which examines the embryonic and fetal origins of chronic diseases, including endocrine–metabolic, immune–allergic, neuropsychiatric, and oncological conditions. In our opinion, DOHaD may be the only theory that fully explains the current epidemiological transition, which appears to be driven by environmental and developmental factors rather than strictly genetic causes. However, to fully grasp this paradigm shift, it is essential to first review key advances in biology and, more specifically, molecular genetics, made over the past two decades.

## 8. Phylogenesis and Ontogenesis: The Role of Genetics and Epigenetics

**Figure 5:**
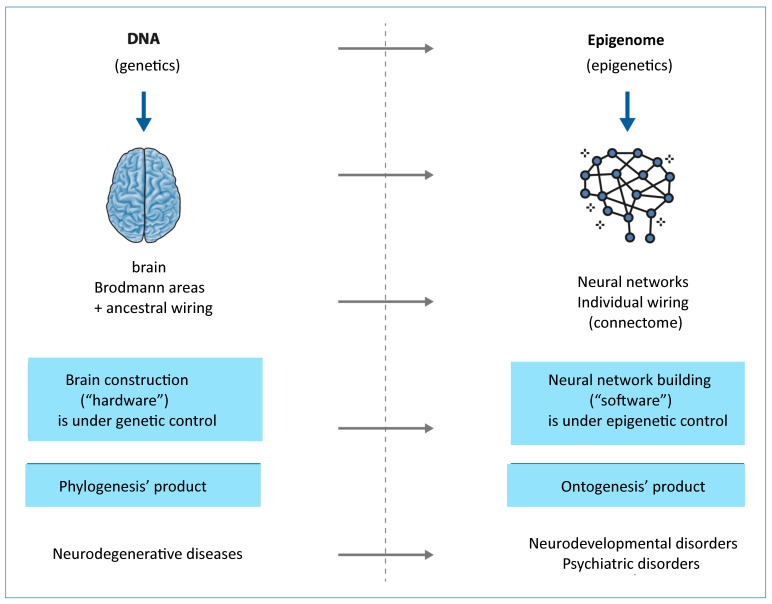
Brain (Brodmann areas + ancestral wiring) is the result of phylogenesis and is almost identical in all members of our species: its development is planned in DNA, which, in its turn, is nearly identical in all of us. Neural networks are the product of interactions between information coming from the environment and the neuronal epigenetic software of everyone during fetal ontogenesis and in the early years of life, resulting in individual wiring (marked nodes).

Based on the points outlined so far, it is clear to us that a radical paradigm shift has been observed from linear to multidimensional systems biology, and this new perspective should be considered in the understanding of health and disease. We should consider the complexity of the feedback loop interactions among the many different molecules involved in any cellular pathway (nucleic acids, proteins, lipids, metabolites, etc.), but most of all, regarding this complex network—which is still a huge challenge for deep learning and AI—we should consider that, beyond nodes, the dynamics of the edges are fundamental. And this dynamic is shaped by the lifespan’s continuous interchange with environmental factors. This shift is also underway in developmental biology, biomedicine, and evolutionary biology. Given the complexity and vast scope of this topic, a comprehensive discussion is beyond the scope of this text. However, we recognize the importance of highlighting some fundamental changes in the dominant model, which impact both evolutionary biology and, consequently, phylogenesis, as well as developmental biology, and thus individual ontogenesis. In our view, without acknowledging these shifts, it would be impossible to fully understand ongoing developments in human health, particularly in the study of neurodevelopmental, neuropsychiatric, and neurodegenerative disorders. The Soviet biologist and geneticist Theodosius Grigorievich Dobzhansky, one of the key figures in modern neo-Darwinian synthesis, famously stated that “nothing in biology makes sense except in the light of evolution”. This statement, when applied to biomedicine, proves particularly insightful. Our bodies are the product of two closely interconnected evolutionary processes: phylogenesis, which refers to the branching evolutionary history of different species, and ontogenesis, which encompasses the biological processes that drive the development of an individual organism—from zygote to embryo, fetus, and ultimately, adult. For a long time, the connection between phylogenesis and ontogenesis was encapsulated by Ernst Haeckel’s famous statement: “Ontogenesis is a recapitulation of phylogenesis”. According to this idea, ontogenesis was thought to summarize the entire phylogenetic process during early stages of life. However, this Recapitulation Theory or Fundamental Biogenetic Law has been widely debated, heavily criticized, and ultimately rejected in its original form. In the 1990s, theorists of Evolutionary Developmental Biology (Evo-Devo) [87] renewed interest in embryology and developmental biology by revisiting the relationship between ontogenesis and phylogenesis from a different perspective. They proposed that certain molecular events and functional changes in the regulation of genes controlling embryonic development could play a decisive role in evolution—potentially leading to the emergence of new phenotypic traits that could be inherited by future generations. According to some researchers, these theories challenge the traditionally dominant role of natural selection by acknowledging, on the one hand, the genome’s intrinsic capacity for reactive–adaptive modifications, and on the other hand, the potential influence of environmental factors in driving evolutionary changes. This influence is particularly significant during the early stages of embryonic and fetal development, when epigenetic plasticity is at its peak. Here, it is both sufficient and necessary to summarize the key aspects of this conceptual shift, particularly in relation to human brain evolution. This includes its rapid development during fetal and neonatal stages and the crucial role of environmental information. Within this new framework, such factors are seen as playing a far more significant role—not only in influencing changes but also in actively shaping and instructing the entire system, rather than merely selecting from random variations. Much like DNA, the fundamental structure of the brain is nearly identical in all individuals of our species. The so-called 52 cortical areas—first described by German neurologist Korbinian Brodmann nearly a century ago—are essentially the same in both morphology and function across all humans. Furthermore, growing evidence suggests that even the major neuronal networks connecting these areas form an inherited, ancestral wiring system (see Figure 5) that we all share. This ancestral wiring links the cortical regions to one another as well as to the oldest parts of the central and peripheral nervous systems—structures that represent a deeply conserved part of the nervous system, shared not only with mammals but also, at least in basic organization, with reptiles and even, to some extent, with Platyhelminthes (flatworms). At the same time, each individual also possesses a unique wiring pattern—an individual connectome—that is specific to the telencephalon, particularly the cortex. This network consists of trillions of synaptic connections that begin to form in the final months of fetal development and continue shaping the brain throughout early postnatal life. These neuronal networks lay the foundation of individual identity and are primarily epigenetically instructed and modulated during ontogenesis, rather than being strictly genetically predetermined and transmitted through phylogenesis. Recent findings suggest that at least some aspects of what could be described as a “family” connectome—neural connectivity patterns passed from parents to offspring—may be heritable. However, it is still too early to determine the full implications of this discovery. If confirmed, it could suggest the existence of a previously unknown, transgenerational, and unexpectedly rapid mechanism for transmitting behavioral phenotypes [88], potentially revolutionizing our understanding of neurodevelopmental inheritance.

Returning to the initial metaphor, we can summarize our perspective by saying that just as DNA serves as a species-specific molecular hardware—the product of millions of years of molecular coevolution (phylogenesis)—the brain can also be considered a species-specific hardware. This brain hardware, shaped by millions of years of evolution, seems likely the result of an instructive–constructive co-evolution rather than a purely selective process. It is nearly identical across all humans and highly similar across primates in terms of structure and basic wiring. Similarly, from our perspective, just as the epigenome functions as an individual molecular software, primarily shaped during embryonic and fetal development in response to environmental signals transmitted through the mother, so too do neuronal networks form the individual connectome. These networks arise mainly from instructive-constructive processes, rather than purely selective ones. They develop with astonishing speed and complexity, primarily during embryonic and fetal ontogenesis, under epigenetic rather than genetic control. This process occurs in response to diverse and complex environmental signals received through the mother and continues throughout life—albeit at a progressively slower rate and with decreasing complexity. If the brain represents a relatively stable hardware, shared across the species and only partially modifiable, then neuronal networks—and, by extension, our mind and psyche—constitute an extraordinarily complex and dynamic software, capable of continuous self-modification throughout life. This concept lies at the heart of psycho-neuroplasticity. This suggested paradigm shift could reshape the entire field of biology—molecular, evolutionary, and developmental—and, consequently, the biomedical sciences of the coming decades. On one hand, the genetic program encoded in DNA—and thus the physiological traits characteristic of our species—remains relatively stable. On the other hand, the dramatic chemical and physical transformations of our environment, caused by human activity in just a few decades, are inducing epigenetic stress. Additionally, but no less significantly, the cultural and structural forms of systematic social violence and injustice (poverty, wars, gender discrimination, racism, alcohol-drug-device addictions, employment-instability, hate speech, violent content perfusion by TV, social media, video games, etc.) determine high psychological–emotional–physical stress or traumas as evidenced by the literature [25,28,89,90,91,92,93]. In our understanding, this stress is generating genuine genomic variability, affecting not only the most exposed or stressed tissues (where it can contribute to carcinogenesis) but, more critically, developing organisms. Due to their heightened epigenetic and genomic plasticity, less differentiated cells—such as embryonic cells and gametes—are particularly vulnerable. If, in the coming years, it will be confirmed that the environment profoundly shapes our phenotype—both in physiological and pathological ways—through continuous reactive–adaptive modifications of our epigenome; and if research ultimately will confirm that at least some of these genomic modifications—especially those occurring during embryonic and fetal development, as suggested by Evo-Devo theories—are heritable across generations, then medicine will also have to acknowledge the fundamental role of the environment in which one grows up. In particular, it will need to recognize the profound impact of the rapid, human induced chemical and physical transformations of the ecosphere—including the atmosphere, hydrosphere, lithosphere, biosphere, and food chains—that have occurred within just a few decades and the results of (in)human policies in a planetary society, whose new generations seem to show the correlated global degree of disease.

In this perspective, when narrowing the focus of this study to its primary subject, the rapid and continuous rise in neurodevelopmental and major depressive disorders should be understood as a direct consequence of increasingly early and widespread exposure of embryos, fetuses, and young children to potentially epigenotoxic environmental factors. These disorders, particularly those with a rising incidence, should be viewed as conditions affecting the connectome and epigenetic software—and, as such, they should be recognized as preventable and reversible, rather than the outcome of irreversible damage to the genetic or cerebral hardware. With this in mind, the next step is to briefly examine the key risk factors and environmental stressors that appear to be correlated with the observed burden and reported increased rates of the “silent pandemic” of neurodevelopmental disorders.

## 9. Risk Factors

Growing research indicates that certain critical periods of fetal and early childhood development (windows of exposure) are characterized by heightened sensitivity to environmental stimuli and information. Neurodevelopment is also marked by sensitive periods, heightened epochs of brain plasticity that extend from conception through early childhood. During these periods, the developing central nervous system (CNS) exhibits profound sensitivity to environmental influences, which effectively shape neural circuits and determine structural and functional aspects of brain and behavior for the lifespan. This process, often referred to as developmental programming, confers either risk for or resilience to later psychopathology [94]. From an epigenetic perspective, particular attention must be paid to late gestation and early childhood, specifically the first 1000 days of life. During this period, developmental plasticity is at its peak, and the body undergoes epigenetic programming of its tissues, organs, and—most importantly—its connectome. Epigenetic mechanisms are highly susceptible to environmental stresses and act as the core molecular link between early-life exposure and subsequent neurodevelopmental outcomes [95]. The susceptibility of epigenetic patterns, being more plastic than the genome sequence itself, makes them a critical determinant of disease risk and etiology. This concept is central to the Developmental Origins of Health and Disease (DOHaD) hypothesis, which posits that experiences during fetal and neonatal periods can induce stable alterations in the epigenome of the offspring, thereby programming long-term health and disease risk. This theory suggests, in fact, that environmental influences during the earliest stages of life can trigger reactive-adaptive and predictive changes in the fetal genome—a process known as fetal programming. These changes, in turn, can have long-term consequences not only for the individual directly affected but also for future generations [86].

Recent findings highlight how a broad spectrum of maternal and environmental factors converge upon the fetal epigenome to program adverse neurodevelopmental trajectories. Those factors include chemical pollutants, neurotoxic agents, maternal inflammation and immune states, metabolic disorders (such as Gestational Diabetes Mellitus and obesity), maternal–fetal stress, complications of premature birth, and the epigenetic risks associated with advanced parental age. Several studies highlight the specific molecular pathways linking these exposures to NDDs such as ASD, SZ, and ADHD.

### 9.1. Environmental Exposures and Epigenetic Neurotoxicology

Among the potential pathogenetic mechanisms involved, a distinction must be made between direct neurotoxic effects—as seen with certain substances such as pesticides and heavy metals (notably lead and mercury)—and more complex epigenetic mechanisms, which, by definition, are particularly significant in early life when developmental plasticity is at its highest. The brain remains highly vulnerable to epigenotoxic factors throughout the entire fetal period and the first two years of life, coinciding with the critical period of neuronal network formation, or connectome development. For decades, it has been established that early exposure to lead, mercury, arsenic, toluene, PCBs (polychlorinated biphenyls), and PAHs (polycyclic aromatic hydrocarbons) can cause serious brain damage. Numerous studies have linked the sharp increase in neurodevelopmental disorders in recent years to the widespread environmental release of these substances into the air, groundwater, and food chains [96,97,98]. It is crucial to emphasize that the near-ubiquitous presence of synergistic pollutants—including tens of thousands of synthetic molecules and byproducts from thermochemical processes such as vehicular emissions and waste incineration near urban centers—is an entirely unprecedented phenomenon in human history. In this context, it is important to recognize that while each organ and tissue has a distinct embryonic–fetal window of exposure, during which its sensitivity to stress and toxins is at its peak, the brain remains highly vulnerable to epigenotoxic factors throughout the entire fetal period and the first two years of life. This prolonged hypersensitivity phase coincides with the critical period of neuronal network formation, or connectome development [99]. Lead is one of the most extensively studied pollutants. Its neurological damage has been well documented, even when exposure occurs at infinitesimal levels. It is known to persist in maternal adipose and bone tissues for decades, readily crosses the placental barrier, and penetrates the blood–brain barrier [100]. While lead concentrations in urban air have significantly decreased, it remains a widespread pollutant—not only in food chains, but also in indoor environments. Consistent research has been performed in recent years to provide sustainable approaches to reduce lead contamination, such as microbial-assisted phytoremediation, which could possibly be a viable option to ensure a safe food production system [101]. Another highly neurotoxic heavy metal is mercury, which disrupts neuronal differentiation, myelination, and synaptogenesis [102]. Despite its known toxicity, mercury remains a pervasive environmental pollutant, primarily as a waste byproduct of thermochemical processes. Furthermore, combined exposure to metals like manganese, mercury, and lead could worsen neurotoxic effects [103]. A critical finding concerns the interaction between neurotoxic metals and psychosocial stress. Recent human studies found that prenatal exposure to lead and mercury, when concurrently associated with psychosocial stress, resulted in dysregulated maternal salivary cortisol. This convergence of chemical and psychological stressors is hypothesized to disrupt the hypothalamic–pituitary–adrenal (HPA) axis function during pregnancy. The molecular mechanism involves increased placental glucocorticoid receptor (NR3C1) DNA methylation, effectively “silencing the brake” on the HPA axis and programming the offspring for hyper-responsive cortisol regulation and subsequent neurobehavioral risk, cognitive problems, and later life stress vulnerability [104]. The hypothesis of a link between early environmental exposure to neurotoxic agents and the rising prevalence of neurodevelopmental conditions (including ASD) is increasingly understood within the framework of the DOHaD theory [95].

Ubiquitous Pollutants and EDCs: Prenatal exposure to ambient air pollution, which is ubiquitous, is documented to adversely affect cognitive and psychomotor capabilities in children [105]. Polycyclic aromatic hydrocarbons (PAHs) and Bisphenol A (BPA) are recognized endocrine-disrupting chemicals (EDCs) that cross the placenta [106]. Maternal exposure to BPA results in postnatal changes in DNA methylation status and altered expression of specific genes in offspring. PAHs have been shown to induce epigenetic modifications by disrupting LINE1 methylation in mother–child cohorts, suggesting interference with gene expression critical for genomic stability and healthy brain development. PAHs are also known to be immunotoxic, affecting the expression of pro-inflammatory cytokines such as tumor necrosis factor-alpha and interleukin-1 beta.

Pesticides: The widespread use of pesticides, including newer classes like neonicotinoids and pyrethroids, is increasingly linked to neurotoxic effects that contribute to neurodevelopmental disorders, even at low exposure levels derived from food consumption. Recent reviews synthesize evidence suggesting that the resulting neuropathology involves the dysregulation of the gut–brain axis, neuroinflammation, and significant epigenetic modulation [107]. For decades, it has been known that in mammals, prenatal exposure to organochlorine pesticides, such as dieldrin and lindane, impairs synaptic function, leading to a decrease in the expression, conformation, and binding ability of the GABA(A) receptor in the brainstem—an essential receptor for postsynaptic neurons [108]. A recent study indicates the effects found after acute exposure to DDT, endosulfan, dieldrin, and lindane occur at concentrations close to, or even below, human internal exposure levels, highlighting the importance of further monitoring human exposure to organochlorine insecticides [109].

The effects of prenatal exposure to chlorpyrifos on cognitive and motor development were assessed in a cohort of 254 children over the first three years of life, based on chlorpyrifos levels in umbilical cord plasma. The findings, published in *Pediatrics* already in 2006 [110], revealed that by age three, children with high exposure had significantly lower scores in assessments of motor and cognitive abilities compared to those with lower exposure levels. Over time, these mental and motor impairments worsened. These outputs had been recently confirmed and associated with prenatal exposure to air pollution in the first and second trimesters [105]. Furthermore, by the age of three, highly exposed children were found to be at a greater risk of being diagnosed with ASD and ADHD. Recently, epidemiological relationships were reported for low-level pesticide exposure and ADHD and/or ASD, further supporting the hypothesis that pesticide exposure at levels that do not cause acute toxicity may be among the multifactorial causes of ADHD and ASD [111]. The hypothesis of a link between early environmental exposure to neurotoxic agents and the rising prevalence of autism spectrum disorders—as well as broader neurodevelopmental, neuropsychiatric, and neurodegenerative conditions—is still debated, even if there is a growing body of supporting evidence in the literature over the past twenty years [112].

### 9.2. Maternal Inflammation, Autoimmunity, and Psycho-Neurotoxicity

Given the importance of vulnerability windows during embryo–fetal development, significant attention has been directed toward the potential impact of maternal immune activation (MIA) during pregnancy. Maternal immune status and inflammation represent potent intrinsic environmental signals that dramatically impact fetal neurodevelopment. A Danish cohort study investigated the association between maternal infections requiring hospitalization during pregnancy and autism diagnosis [113]. The findings indicated an increased risk of autism among children whose mothers experienced viral infections in the first trimester or bacterial infections in the second trimester. A separate study examined the possible link between maternal influenza infection and ASD risk in children. The results showed that influenza during pregnancy itself was not associated with increased risk, but febrile episodes—particularly when untreated with anti-fever medication—were linked to a higher ASD risk [114]. Beyond the effects of infection-induced immune activation, researchers have also explored the role of maternal autoimmunity. Mothers with chronic autoimmune conditions, such as Systemic Lupus Erythematosus (SLE) or Rheumatoid Arthritis (RA), have heightened systemic inflammation [115]. The resulting autoantibodies and cytokines cross the placenta, directly altering fetal neural circuits and synaptic function. Converging neuroimmune and metabolic pathways, when disrupted in utero, substantially alter the developmental trajectory of the brain and increase the likelihood of ASD. Such interruptions leading to developmental changes can be triggered by immune activation from environmental sources of infection and pollution, as in cases of autoimmune disease or cerebral folate deficiency [116]. There is also evidence that parental autoimmune diseases are associated with ASD in offspring [117,118], suggesting that there may be common pathogenic, developmental mechanisms related to autoimmunity that are associated with the etiology of ASD [119]. The primary molecular pathway driving neurodevelopmental illness following MIA involves the epigenetic impairment of -aminobutyric acid (GABA) synthesis. Specifically, MIA leads to increased 5-methylcytosine (5-mC) and 5-hydroxymethylcytosine (5-hmC) modifications—a condition of hypermethylation—at the promoter regions of the GAD1 and GAD2 genes. Since the encoded glutamate decarboxylases are responsible for GABA production, their epigenetic silencing causes altered synaptic responses in early life and contributes to impaired cognitive and social responses, consistent with the phenotypes observed in ASD [120]. Another aspect of maternal immune influence involves the production of autoantibodies against fetal CNS antigens during intrauterine life. Studies have identified autoantibodies targeting brain antigens in both children and adults with ASD. The type of ASD associated with the presence of maternal autoantibodies has been referred to as maternal antibodies related to ASD (MAR ASD). This mechanism involves IgG crossing the placenta and binding to fetal brain proteins, disrupting neural development, synaptic function, and neurotransmitter balance [121]. The primary antigens identified in the panel of specific neurodevelopmental proteins include Lactate Dehydrogenase A/B (LDHA/B), Stress-Induced Phosphoprotein 1 (STIP1), Collapsin Response Mediator Proteins 1 and 2 (CRMP1, CRMP2), neuron-specific enolase (NSE), Cypin, Guanine Deaminase (GDA), and Y-box-binding protein (YBOX-1). Reactivity to specific combinations of these antigens, particularly LDH, STIP1, and CRMP1 and/or Cypin, shows high clinical significance with over 99% specificity for autism risk and an Odds Ratio (OR) of 24.2. Overall, exclusive reactivity to specific antigen patterns was found in 23% of mothers of children with ASD compared to 1% of controls [122]. Studies screening archived mid-pregnancy blood samples confirm the gestational presence of these fetal brain-reactive antibodies, specifically those recognizing the 39 kDa and 73 kDa antigens, validating their role as an etiological risk factor [123]. Further, the same antigens have been recently confirmed as potential biomarkers of ASD and ASD comorbidities [124]. In a recent study, a new model of maternal aAb exposure rat was developed, and it has shown alterations in behavior, brain structure, and neurometabolites, reminiscent of findings observed in clinical ASD [125].

### 9.3. Maternal Metabolic Disorders and Lifestyle

**Figure 6:**
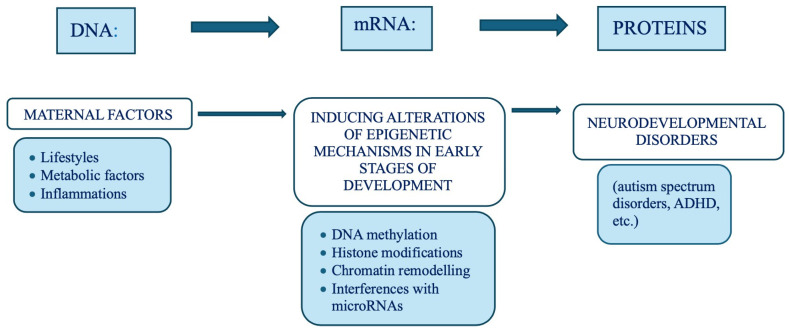
Neurodevelopmental disorder correlation with maternal metabolic factors.

Extensive scientific evidence suggests that maternal metabolic disorders can disrupt epigenetic mechanisms, leading to neurodevelopmental and metabolic abnormalities in offspring. Multiple studies have shown that fetuses exposed to altered maternal metabolic conditions like Gestational Diabetes Mellitus, maternal obesity, and Congenital Hypothyroidism have a higher likelihood of developing autism spectrum disorders, developmental delays, ADHD, eating disorders, and psychotic disorders later in life. Maternal lifestyle factors such as smoking, psychotropic substance consumption, alcoholism, and psychosocial stress are reported to be critically associated with abnormal neurological syndrome in childhood. Smoking and alcohol consumption during gestation are the most detrimental habits that have been shown to affect language, speech, hearing, and cognitive development in offspring [Figure 6]. Consequently, early treatment of metabolic disorders, therapeutic epi-drugs—ideally before conception—and information/sensitization campaigns among vulnerable social conditions are crucial preventive measures to mitigate or reverse potential adverse effects and abnormalities in the brain on fetal development, as epigenetic marks are plastic and reversible in nature [126,127,128].

### 9.4. Maternal–Fetal Stress and Its Psycho-Neurotoxic Effects on the Fetus

Prenatal maternal stress, encompassing psychological distress, anxiety, depression, and acute traumatic events, has profound psycho-neurotoxic effects, robustly increasing the risk of neurodevelopmental challenges in offspring. Persistent prenatal maternal–fetal stress plays a critical role in altering the epigenetic programming of key brain regions and the hypothalamic–pituitary–adrenal (HPA) axis in the fetus. The biological mechanism linking maternal stress to fetal outcomes is highly conserved and centered on the maternal-placental-fetal axis, resulting in HPA axis dysregulation, placental gene expression changes, epigenetic modifications, and neuroinflammatory responses [129,130]. In particular, it has been observed that glucocorticoids, the downstream effectors of stress, are known to alter DNA methylation in the fetal hippocampus, a region vital for memory and emotion regulation. This alteration serves to “prime” future stress responses in the offspring, leading to increased vulnerability to emotional dysregulation, anxiety, and psychiatric disorders. Furthermore, research utilizing chronic stress models has identified the dynamic accumulation of the novel epigenetic mark N(6)-methyladenine (6 mA) in the mouse prefrontal cortex. Genes bearing stress-induced 6 mA changes significantly overlap with loci associated with depression, SZ, and ASD [131].

### 9.5. Premature Births and Placental Inflammation: Psychoneurotoxic Effects on the Fetus

Today, more than one in ten babies are born prematurely, with a 15% of all preterm births occurring at “very preterm”, that is, less than 32 weeks of gestation. Preterm birth is the leading cause of neonatal mortality and is associated with long-term physical, neurodevelopmental, and socioeconomic effects [132]. The placenta is a critical interface, and its disruption—such as that associated with preterm premature rupture of membranes (pPROM) and subsequent inflammation—directly influences neonatal and long-term neurodevelopmental outcomes. Placental inflammation, whether sterile or pathogenic, creates a pro-inflammatory intrauterine environment that disrupts cellular integrity, impairs nutrient exchange, and amplifies inflammatory insults to the fetus. This inflammatory cascade induces epigenetic changes that mediate adverse neurodevelopmental trajectories. The resulting dysfunction extends its impact to the developing brain by activating microglia—the brain’s resident immune cells [133]. Nevertheless, prematurity itself is a significant risk factor for neurodevelopmental disorders, as the final stages of brain maturation occur in an environment drastically different from the womb. To grasp the magnitude of this impact, it is important to consider the extraordinary pace of brain growth during fetal development and early childhood. Neurons are added at an estimated rate of 250,000 per minute, with even higher proliferation rates in the final two months of gestation when a substantial portion of brain development takes place. At birth, the cerebral cortex alone contains approximately 30 billion neurons, each forming around 2500 synapses. By the sixth month of gestation, the brain’s surface remains relatively smooth, as the major gyri form progressively as space for growth becomes more constrained. Consequently, in “very preterm” infants, this critical stage of brain development unfolds under conditions vastly different from those that have been biologically “natural” for millions of years.

### 9.6. Parental Age and the Risk of Autism and Schizophrenia

Advanced parental age (APA) is one of the most consistently identified perinatal risk factors for neurodevelopmental disorders, specifically ASD and SZ. Studies suggest that children born to parents over the age of 35 face a higher risk of autism, with the likelihood increasing as parental age advances [134]. APA is associated with specific neurobiological and behavioral phenotypes in offspring, including higher anxiety, repetitive behaviors, and social–communication deficits. At the molecular level, genome-wide methylation analysis links APA to differential methylation in humans, implicating key neurodevelopmental genes such as CDH9 and ZNF266. Another key mechanism involves microRNA dysregulation. In particular, miR-132 and miR-134, critical for neuronal morphogenesis and dendritic growth, are differentially regulated depending on APA status. Critically, the epigenetic effects from the paternal line can be transmitted because certain imprinted genes, such as the one encoding miR-134, escape the traditional epigenetic reprogramming that typically occurs after fertilization. This persistence allows the paternal methylation pattern to be inherited, transferring paternal age effects onto the offspring’s behavior and brain development, particularly affecting fronto-hippocampal connectivity [135]. Scientific evidence suggests that environmental and maternal factors induce neurodevelopmental risk by exploiting a limited number of critical epigenetic regulatory pathways. The fetal epigenome serves as an integrated receiver, processing inputs from external pollutants, internal metabolic states, immune signals, and psychosocial stress. Two hubs stand out as key targets for this programming:

1. The HPA Axis/Glucocorticoid Signaling: Targeted by both neurotoxic metals (lead and mercury) and psychosocial stress via hypermethylation, which programs anxiety and stress vulnerability.
2. GABAergic Signaling: Targeted by maternal inflammation and infection (MIA) via hypermethylation of the genes, leading to synaptic dysfunction and ASD-like phenotypes.

### 9.7. The Adolescent Brain: A Critical Period of Development

Adolescence is a crucial stage of brain development, marked by significant structural and functional changes that shape cognitive abilities, emotional regulation, and behavior. During this period, the brain remains highly plastic, making it particularly sensitive to environmental influences, learning experiences, and social interactions. One of the most notable transformations occurs in the prefrontal cortex, the region responsible for decision-making, impulse control, and rational thinking. Although the brain reaches approximately 80% of its adult size by the age of two, it still contains an excessive number of synaptic connections—around 10,000 to 12,000 per neuron. Many of these connections are gradually refined through synaptic pruning, a process that strengthens frequently used neural pathways while eliminating weaker ones. This selective process, governed by apoptosis, peaks during adolescence and plays a fundamental role in cognitive and emotional maturation. Disruptions in these neurodevelopmental processes can have lasting consequences. While some neurodevelopmental disorders stem from impaired synaptogenesis (the formation of synaptic connections) and abnormalities in neural network formation, dysregulated pruning mechanisms are increasingly linked to the onset of major neuropsychiatric disorders, particularly SZ. In an extraordinary collaborative research, recently published in *Nature Medicine*, a promising neuropsychopathological factor has been identified across externalizing and internalizing symptoms, using multitask connectomes, bridging multidimensional evidence from behavioral, neuroimaging, and genetic substrates. As a suitable biomarker of psychiatric comorbidity, it was able to recognize a reproducible and general neural basis underlying symptoms of multiple mental health disorders. It is remarkable that it has been found enriched in the frontal and parietal lobes, indicating atypical trajectories of neural circuit maturation and inhibition of the maturational process of synaptic pruning and synapse stabilization, with consequences on gray matter volume and emotional behavior. Both synaptogenesis and pruning are strongly influenced by epigenetic regulation, highlighting the intricate interplay between genetic predisposition and environmental factors in shaping adolescent brain development [4].

## 10. Salutogenic Factors

The first 1000 days of life provide an unparalleled opportunity to shape lifelong health outcomes. By reducing exposure to harmful environmental factors and promoting positive epigenetic influences, we have the potential to dramatically improve the health of millions of children and future generations. Since the final stages of embryo–fetal development are particularly sensitive to environmental inputs—where epigenetic regulation often outweighs genetic programming—this period is especially vulnerable to environmental influences. Given its crucial role in synaptogenesis and neural network formation, it is increasingly vital for the scientific community to prioritize research not only on risk factors during the critical windows of development, but also on positive factors that can promote, prevent, revert maladaptive and malaise into safe and healthy neurodevelopment.

By identifying and mitigating maternal–fetal exposure to environmental hazards, targeted primary prevention strategies need to be globally implemented to reduce the incidence of these disorders. As demonstrated by research in the field of animal studies, the epigenetic state of a gene can be established through the process of behavioral programming [136]. Increased licking and grooming of pups, as well as arcuate lactation, alter the epigenome of the offspring in a region of the hippocampus associated with the glucocorticoid receptor gene. The quality of maternal care has been shown to affect anxiety-related behaviors and the epigenetic programming of glucocorticoid receptor gene expression. This finding indicates that alterations in environmental conditions during early development can exert a significant influence on neurological characteristics associated with neurodevelopmental disorders, potentially increasing their severity or manifestation. It is evident that appropriate environmental enrichment during the first thousand days of life significantly influences epigenetic modifications related to cognitive deficits and neural plasticity, as well as cognitive behavioral therapy in childhood and adolescence [46]. The transition in those fields from animal models to humans is absolutely not obvious, due to the complexity of real environments in which humans live vs. laboratory standard conditions, and the difficulty of standardizing “enriched” environments for reproducibility and evaluation [47]. Nevertheless, interventions such as parental education, psychological support, breastfeeding promotion, reduction in environmental toxins, meditation, proper educational approaches, and psychosocial stimulation have been shown to positively shape the child’s epigenetic profile [48,49]. For instance, programs that promote skin-to-skin contact, responsive caregiving, and stress reduction in NICUs (neonatal intensive care units) have demonstrated encouraging results in enhancing neurodevelopmental outcomes, particularly in preterm infants, whose epigenomes are especially malleable. It is imperative to recognize the significance of the broader social, cultural, and environmental context influencing early epigenetic programming [48,49,50]. A range of socio-educational–economic factors—including maternal education, access to healthcare, and exposure to systemic stressors such as discrimination and housing instability—has been shown to impact developmental outcomes through epigenetic pathways. It is therefore crucial that policies aimed at promoting maternal mental health, food security, awareness, educated and clean-living environments during pregnancy and infancy consider all these factors not only as social imperatives but also as biological necessities.

## 11. Implications for Research and Prevention

If neurodevelopmental risk emerges from interactions among genetic susceptibility, developmental timing, and modifiable exposures, then progress depends on study designs and analytic strategies that can separate correlation from causation to identify not just negative but positive markers and indicators. Key priorities include the following:

→ Educational programs to increase the awareness and knowledge of parents, students, and educators, to be validated and suggested as best practice;
→ Educational programs for schools to promote salutogenesis, to be validated and suggested as best practice;
→ Prospective pregnancy, birth cohorts, and follow-ups with diverse exposures to risk or salutogenic factors, measured and evaluated repeatedly across sensitive windows;
→ In animal models, puppies from the same litter, born to mothers with different types of stress and typical epigenomes, could be raised in a neutral or enriched environment to assess the reversibility of epigenetic marks of the negative conditions at birth;
→ Integrated multi-omics (e.g., methylation, transcriptomics, and metabolomics) with careful attention to tissue specificity (placenta, cord blood, amniotic fluid, and, where feasible, brain-relevant models) to evaluate the phenotypes correlated with epigenetic marks and different exposures;
→ Triangulation using complementary causal-inference approaches (e.g., sibling comparisons, negative controls, and mediation analyses where assumptions are met);
→ Replication and rigorous control of batch effects in epigenetic studies;
→ Translational bridges that test mechanisms in diverse experimental systems (animal models, iPSC-derived neurons/organoids);
→ Most urgently, none of these perspectives of research would be feasible if salutogenic and positive environmental factors are not suitable, which means prevention strategies and policy interventions are needed.

## 12. Concluding Remarks: Epigenetics, the Bridge Between Genes and Environment, and the Field of Human Responsibility

Rather than a fixed, deterministic genetic code, the genome is now understood to be highly responsive to environmental inputs, allowing continuous and bidirectional communication between genetic material and the outside world. This shift in perspective redefines the role of the environment, which is now seen as a constant flow of information that can reshape gene expression patterns and, particularly during early development, even reprogram the epigenome itself. Environmental factors such as nutrition, toxins, stress, and lifestyle choices play a far more significant role than previously thought, influencing both physiological adaptation and disease susceptibility through epigenetic mechanisms. The alarming rise in neurodevelopmental and neuropsychiatric disorders is evidently correlated with interferences with the dynamic and sensitive epigenome in the earliest stages of life. The rapid changes in our environment, diet, chemical exposures, personal and social violence, loss of life significance, and lifestyle seem to have outpaced our biological ability to adapt, leading to dysregulated epigenetic programming and increased disease risk. From our perspective, this means that there is an urgent need for a global reflection on how to comply with the WHO definition of health, namely a state of “complete physical, mental, and social well-being and not merely the absence of disease or infirmity”. This definition emphasizes that health is a holistic concept, encompassing mental, social, and spiritual dimensions in addition to physical health, and that enjoying the highest attainable standard of health is a fundamental human right. Even just the suspicion, based on the animal evidence, that our choices of today do not influence just our life but also that of new generations, and the burden of NDD, urges us to demand an unpostponable coherence and responsibility in terms of environmental, economic, educational, and social politics to ensure global public health. The symptomatology of a disorder is partially a reflection of its context, in a continuous and dynamic dialog between the contextual and the biological, the social and the individual. The understanding that in this complex network the edges (i.e., interactions and relationships) are even more important than the nodes (i.e., individual elements) underlines the need for a multifactorial consideration—encompassing epidemiological, socio-neurobiological, and individual clinical-molecular aspects—for person-centered diagnoses in NDDs. Although it is well established that epigenetic changes associated with disease can occur throughout life, the particularly labile nature of the epigenome during early development makes this period especially significant and decisive. Given the exponential increase in consultations for neurodevelopmental problems in pediatrics, we believe it is imperative to raise awareness among stakeholders and promote urgent and concrete interventions in environmental and educational policy to improve early diagnosis, prognosis, and—most importantly—to foster safe and healthy development. In conclusion, research in the field of epigenetics has fundamentally transformed our understanding of how early childhood experiences become biologically embedded. Contrary to earlier views that saw infants as passive recipients of their genetic makeup, current perspectives highlight their dynamic nature: children are active agents whose developmental trajectory is continuously shaped by their physical and social environments. This recognition calls for an integrated and preventive approach to health—one that values not only the absence of risk, but also the proactive creation of nurturing, salutogenic, enriching conditions during the most plastic and vulnerable stages of life. Education to nonviolent and salutogenic environments, meditation, and life-meaningful practices need to be introduced in all school systems, from an early age, as only community-based approaches can ensure public health, equity and democracy, for the wellbeing and safety of present and next generations and for the future of the human species.

### Literature Search and Selection

This is not a systematic review that used predefined methods, but a narrative review conducted by the authors based on their experiences, knowledge, and interpretations to contextualize the topic in its complexity, offering a broad and flexible overview, but also making their perspective explicit. The literature search was performed mainly on PubMed/MEDLINE, Scopus, and Web of Science for English-language articles (2000–2025), prioritizing systematic reviews, meta-analyses, large cohort studies, and mechanistic studies relevant to epigenetics, risk factors, and neurodevelopment.

## Figures and Tables

**Table 1:** Overview of NDDs’ epigenetic signatures [1,64,65,66,67,68,69,70].

NDD	Epigenetic Mechanism	Specific Signature (Gene/Locus/Mark)	Type & Direction of Change	Observed Effect/Implication
**ASD**	DNA Methylation	Global DNA	Hypomethylation (in peripheral tissues)	Correlates with disease severity; potential biomarker
	DNA Methylation	*NCAM1*, *NGF*, *ST8SIA2*	Increased methylation	Altered neuronal cell adhesion, growth factor signaling
	DNA Methylation	*OXTR*, *HTR4*, *TGFB1*	Hypomethylation	Altered social behavior, serotonin signaling, immune response
	DNA Methylation	*ESR2*	Hypermethylation	Associated with symptom severity
	DNA Methylation	*MECP2*, *OXTR*, *HTR1A*, *RELN*, *BCL-2*, *EN-2*	Sex-related methylation differences	Influences sex-specific ASD phenotypes
	Histone Modification	*ASH1L*, *KDM5B*, *KMT2C*, *SETD2*, *SETDB1*	Mutations/Dysregulation	Affects NPC proliferation/differentiation, WNT signaling, cortical malformations
	Non-coding RNA	*RAY1/ST7*, *ST7OT1–3*	Rare variants	Implicated in ASD onset
	Non-coding RNA	*lnc-NR2F1*, *SYNGAP1-AS*, *MSNP1AS*	Upregulation	Affects neuronal maturation, RHOA pathway, cytoskeleton dynamics
**ADHD**	DNA Methylation	*DRD4* promoter, *LIME1*, *SPTBN2*, *ZNF814*, *ELF4*, *OR6K6*, *APOB*, *LPAR5*, *NET* promoter	Altered methylation patterns (hyper/hypo)	Associated with ADHD symptoms, persistence, executive function
	Histone Modification	H3K4Me3 (general), HDACs 2/3	Dysregulation (e.g., decreased acetylated H3, increased HDACs)	Affects synaptic plasticity, neuronal circuits, gene transcription
	Non-coding RNA	*KDM4A-AS1*, *LINC02497*, *LINC02060*, *TMEM161B-AS1*, *LINC01288*, *LINC01572*, *MEF2C-AS1*, *LINC00461*	Associated with risk (non-coding variants)	Pleiotropic effects across psychiatric disorders (*LINC00461*); influences gene expression
	Non-coding RNA	*let-7d*, *miR-384-5p*	Abnormal expression	Affects learning and memory, neuronal function
**ID**	DNA Methylation	*DNMT3B* (ICF syndrome), *DNMT3A* (TBRS)	Mutations leading to aberrant methylation (hypo/hyper)	Impaired neurogenesis, neuronal differentiation, migration
	Histone Modification	*EHMT1* (Kleefstra syndrome)	Mutations affecting H3K9 mono- and dimethylation	Developmental delay, cognitive impairments
	Histone Modification	*EZH2*, *NSD1* (Weaver syndrome), *NSD2* (Wolf-Hirschhorn syndrome)	Haploinsufficiency/Mutations affecting H3K27me3, H3K36 methylation	Cognitive deficits, neuronal migration defects, growth abnormalities
	Histone Modification	*ATRX* (ATRX syndrome)	Mutations affecting H3K9me3 marks	Intellectual disability, cognitive defects, dysregulated DNA methylation
	Non-coding RNA	*FMR1* gene (*FMR4*, *FMR1-AS1*, *FMR5*, *FMR6*), miRNAs (*miR-302*, *miR-125*, *miR-132*)	Dysregulation/Inactivation (FXS)	Impaired neuronal development, synaptic function
	Non-coding RNA	*SNORD116* clusters (PWS), *Ube3a-ATS* (AS)	Deletion/Epigenetic silencing	Contributes to ID phenotypes in PWS and AS
	Non-coding RNA	*lnc-NR2F1*, *FLJ16341*, *LINC00299*	Mutations/Disruption	Affects neuronal maturation, migration, neurodevelopmental delay
	ATP-Dependent Chromatin Remodeling	BAF complexes (*Brg1*)	Dysregulation/Loss of function	Defects in neurogenesis, gliogenesis, abnormal cerebral development
**SZ**	DNA Methylation	*reelin* promoter, *BDNF* gene promoter, *SOX10* gene, *GAD67* enzyme, *RELN* gene	Methylation changes (hyper/hypo)	Abnormal GABA synthesis, myelin development, oligodendrocyte dysfunction
	Histone Modification	H2A.Z	Hyperacetylation	Associated with abnormal gene expression in SZ neurons
	Histone Modification	H3K9	Increased di-methylation (esp. in men)	Associated with elevated GLP and SETDB1 activities
	Histone Modification	H3K4, H3K27	Decreased H3K4 trimethylation, Increased H3K27 trimethylation	Altered chromatin activity, gene repression/activation
	Histone Modification	*GAD67* gene promoter	Reduced H3K4 methylation (in women)	Decreased GAD67 expression, affecting GABAergic function
	Non-coding RNA	MicroRNAs	Differentially expressed	Potential role in SZ pathogenesis

**Table 2:** Estimated prevalence of autism spectrum disorder (2000–2022) among 8-year-old children in the US (ADDM Network of CDS).

Surveillance Year	Birth Year	Number of ADDM Sites Reporting	Combined Prevalence per 1000 Children (Range Across ADDM Sites)	1 in X Children
2022	2014	16	32.2 (9.7–53.1)	1 in 31
2020	2012	11	27.6 (23.1–44.9)	1 in 36
2018	2010	11	23.0 (16.5–38.9)	1 in 44
2016	2008	11	18.5 (18.0–19.1)	1 in 54
2014	2006	11	16.8 (13.1–29.3)	1 in 59
2012	2004	11	14.5 (8.2–24.6)	1 in 69
2010	2002	11	14.7 (5.7–21.9)	1 in 68
2008	2000	14	11.3 (4.8–21.2)	1 in 88
2006	1998	11	9.0 (4.2–12.1)	1 in 110
2004	1996	8	8.0 (4.6–9.8)	1 in 125
2002	1994	14	6.6 (3.3–10.6)	1 in 150
2000	1992	6	6.7 (4.5–9.9)	1 in 150

## Data Availability

No new data were created or analyzed in this study. Data sharing is not applicable to this article. Figures or tables are original creations, except Figure 4, which is adapted from [41] and is published under the Creative Commons Attribution (CC BY) license.

## Abbreviations

NDDs	Neurodevelopmental disorders
ASD	Autism Spectrum Disorder
ADHD	Attention-Deficit/Hyperactivity Disorder
ID	Intellectual Disability
MDD	Major Depressive Disorders
SZ	Schizophrenia
SLDs	Specific Learning Disorders

## References

[1] Reichard J., Zimmer-Bensch G. (2021). The Epigenome in Neurodevelopmental Disorders. Front. Neurosci..

[2] Adamczyk P.M., Shaw A., Morella I.M., More L. (2025). Neurobiology, molecular pathways, and environmental influences in antisocial traits and personality disorders. Neuropharmacology.

[3] Lee S., McAfee J.C., Lee J., Gomez A., Ledford A.T., Clarke D., Min H., Gerstein M.B., Boyle A.P., Sullivan P.F. (2025). Massively parallel reporter assay investigates shared genetic variants of eight psychiatric disorders. Cell.

[4] Xie C., Xiang S., Shen C., Peng X., Kang J.J., Li Y., Cheng W., He S., Bobou M., Broulidakis M.J. (2023). A shared neural basis underlying psychiatric comorbidity. Nat. Med..

[5] Cross-Disorder Group of the Psychiatric Genomics Consortium (2019). Genomic Relationships, Novel Loci, and Pleiotropic Mechanisms across Eight Psychiatric Disorders. Cell.

[6] Bacon E.R., Brinton R.D. (2021). Epigenetics of the developing and aging brain: Mechanisms that regulate onset and outcomes of brain reorganization. Neurosci. Biobehav. Rev..

[7] Horsthemke B. (2018). A critical view on transgenerational epigenetic inheritance in humans. Nat. Commun..

[8] Fernandez-Twinn D.S., Constância M., Ozanne S.E. (2015). Intergenerational epigenetic inheritance in models of developmental programming of adult disease. Semin. Cell Dev. Biol..

[9] Mulligan C.J., Quinn E.B., Hamadmad D., Dutton C.L., Nevell L., Binder A.M., Panter-Brick C., Dajani R. (2025). Epigenetic signatures of intergenerational exposure to violence in three generations of Syrian refugees. Sci. Rep..

[10] Dolinoy D.C., Weidmana J.R., Jirtle R.L. (2007). Epigenetic gene regulation: Linking early developmental environment to adult disease. Reprod. Toxicol..

[11] Hacker K. (2024). The Burden of Chronic Disease. Mayo Clin. Proc. Innov. Qual. Outcomes.

[12] Wang G., Walker S.O., Hong X., Bartell T.R., Wang X. (2013). Epigenetics and Early Life Origins of Chronic Noncommunicable Diseases. J. Adolesc. Health.

[13] Kundakovic M., Champagne F.A. (2015). Early-life experience, epigenetics, and the developing brain. Neuropsychopharmacology.

[14] Kwon E., Kim Y.J. (2017). What is fetal programming?: A lifetime health is under the control of in utero health. Obstet. Gynecol. Sci..

[15] Subramanian M., Wojtusciszyn A., Favre L., Boughorbel S., Shan J., Letaief K.B., Pitteloud N., Chouchane L. (2020). Precision medicine in the era of artificial intelligence: Implications in chronic disease management. J. Transl. Med..

[16] Encode Project Consortium (2012). An integrated encyclopedia of DNA elements in the human genome. Nature.

[17] Shapiro J.A. (2009). Revisiting the Central Dogma in the 21st Century. Ann. N. Y. Acad. Sci..

[18] Balestrieri E., Arpino C., Matteucci C., Sorrentino R., Pica F., Alessandrelli R., Coniglio A., Curatolo P., Rezza G., Macciardi F. (2012). HERVs Expression in Autism Spectrum Disorders. PLoS ONE.

[19] Guffanti G., Gaudi S., Fallon J.H., Sobell J., Potkin S.G., Pato C., Macciardi F. (2014). Transposable elements and psychiatric disorders. Am. J. Med. Genet. Part B.

[20] Dash S., Syed Y.A., Khan M.R. (2022). Understanding the Role of the Gut Microbiome in Brain Development and Its Association with Neurodevelopmental Psychiatric Disorders. Front. Cell Dev. Biol..

[21] Dutton C.L., Maisha F.M., Quinn E.B., Morales K.L., Moore J.M., Mulligan C.J. (2023). Maternal Psychosocial Stress Is Associated with Reduced Diversity in the Early Infant Gut Microbiome. Microorganisms.

[22] Krubitzer L., Kahn D.M. (2003). Nature versus nurture revisited: An old idea with a new twist. Prog. Neurobiol..

[23] Perna R., Harik L. (2017). Nature (Genes), Nurture (Epigenetics), and Brain Development. J. Pediatr. Neonatal Care.

[24] Meaney M.J. (2010). Epigenetics and the Biological Definition of Gene × Environment Interactions. Child Dev..

[25] Penner-Goeke S., Binder E.B. (2024). Linking environmental factors and gene regulation. eLife.

[26] Dolinoy D.C., Das R., Weidman J.R., Jirtle R.L. (2007). Metastable epialleles, imprinting, and the fetal origins of adult diseases. Pediatr. Res..

[27] Kubota T. (2016). Epigenetic alterations induced by environmental stress associated with metabolic and neurodevelopmental disorders. Environ. Epigenet..

[28] Mulligan C.J. (2021). Systemic racism can get under our skin and into our genes. Am. J. Phys. Anthropol..

[29] Jaenisch R., Bird A. (2003). Epigenetic regulation of gene expression: How the genome integrates intrinsic and environmental signals. Nat. Genet..

[30] Mulligan C.J. (2025). Epigenetic age acceleration and psychosocial stressors in early childhood. Epigenomics.

[31] Fraga M.F., Ballestar E., Paz M.F., Ropero S., Setien F., Ballestar M.L., Heine-Suñer D., Cigudosa J.C., Urioste M., Benitez J. (2005). Epigenetic differences arise during the lifetime of monozygotic twins. Proc. Natl. Acad. Sci. USA.

[32] Hannon E., Knox O., Sugden K., Burrage J., Wong C.C.Y., Belsky D.W., Corcoran D.L., Arseneault L., Moffitt T.E., Caspi A. (2018). Characterizing genetic and environmental influences on variable DNA methylation using monozygotic and dizygotic twins. PLoS Genet..

[33] Zenk F., Loeser E., Schiavo R., Kilpert F., Bogdanović O., Iovino N. (2017). Germ line-inherited H3K27me3 restricts enhancer function during maternalto-zygotic transition. Science.

[34] Reik W. (2007). Stability and flexibility of epigenetic gene regulation in mammalian development. Nature.

[35] Drake A.J., Walker B.R. (2004). The intergenerational effects of fetal programming: Non-genomic mechanisms for the inheritance of low birth weight and cardiovascular risk. J. Endocrinol..

[36] Banushi B., Collova J., Milroy H. (2025). Epigenetic Echoes: Bridging Nature, Nurture, and Healing Across Generations. Int. J. Mol. Sci..

[37] Burgio E., Piscitelli P., Colao A. (2018). Environmental Carcinogenesis and Transgenerational Transmission of Carcinogenic Risk: From Genetics to Epigenetics. Int. J. Environ. Res. Public Health.

[38] Webster A.K., Phillips P.C. (2025). Epigenetics and individuality: From concepts to causality across timescales. Nat. Rev. Genet..

[39] Verdikt R., Armstrong A.A., Allard P. (2023). Transgenerational inheritance and its modulation by environmental cues. Curr. Top. Dev. Biol..

[40] Khan Z., El Messiri N., Iqbal E., Hassan H., Tanweer M.S., Sadia S.R., Taj M., Zaidi U., Yusuf K., Syed N.I. (2025). On the role of epigenetic modifications of HPA axis in post traumatic stress disorder and resilience. J. Neurophysiol..

[41] Knudsen T.M., Rezwan F.I., Jiang Y., Karmaus W., Svanes C., Holloway J.W. (2018). Transgenerational and intergenerational epigenetic inheritance in allergic diseases. J. Allergy Clin. Immunol..

[42] Cavalli G., Heard E. (2019). Advances in epigenetics link genetics to the environment and disease. Nature.

[43] Takahashi Y., Valencia M.M., Yu Y., Ouchi Y., Takahashi K., Shokhirev M.N., Lande K., Williams A.E., Fresia C., Kurita M. (2023). Transgenerational inheritance of acquired epigenetic signatures at CpG islands in mice. Cell.

[44] Kachhawaha A.S., Mishra S., Tiwari A.K. (2023). Epigenetic control of heredity. Prog. Mol. Biol. Transl. Sci..

[45] Korolenko A.A., Noll S.E., Skinner M.K. (2023). Epigenetic Inheritance and Transgenerational Environmental Justice. Yale J. Biol. Med..

[46] Wood J.J., Drahota A., Sze K., Har K., Chiu A., Langer D.A. (2009). Cognitive behavioral therapy for anxiety in children with autism spectrum disorders: A randomized, controlled trial. J. Child Psychol. Psychiatry.

[47] Ball N.J., Mercado E., Orduna I. (2019). Enriched environments as a potential treatment for developmental disorders: A critical assessment. Front. Psychol..

[48] Kaliman P. (2019). Epigenetics and meditation. Curr. Opin. Psychol..

[49] Kubota T., Misciagna S. (2022). Biological understanding of neurodevelopmental disorders based on epigenetics, a new genetic concept in education. Learning Disabilities—Neurobiology, Assessment, Clinical Features and Treatments.

[50] Gaudi S., Guffanti G., Fallon J., Macciardi F. (2016). Epigenetic mechanisms and associated brain circuits in the regulation of positive emotions: A role for transposable elements. J. Comp. Neurol..

[51] Yehuda R., Engel S.M., Brand S.R., Seckl J., Marcus S.M., Berkowitz G.S. (2005). Transgenerational effects of posttraumatic stress disorder in babies of mothers exposed to the World Trade Center attacks during pregnancy. J. Clin. Endocrinol. Metab..

[52] Yehuda R., Bierer L.M. (2008). Transgenerational transmission of cortisol and PTSD risk. Prog. Brain Res..

[53] Cusick S.E., Georgieff M.K. (2016). The Role of Nutrition in Brain Development: The Golden Opportunity of the “First 1000 Days”. J. Pediatr..

[54] Katus L., Lloyd-Fox S. (2025). Broadening the lens: How 25 years of prospective longitudinal studies have reshaped infant neurodevelopment in the majority world. Infant. Behav. Dev..

[55] Aristotle (1982). De Generatione et Corruptione.

[56] Aristotle (1908). De Respiratione, Parva Naturalia.

[57] Aristotle (1957). De Vita et Morte Parva Naturalia.

[58] Heard E., Martienssen R.A. (2014). Transgenerational Epigenetic Inheritance: Myths and Mechanisms. Cell.

[59] Morris-Rosendahl D.J., Crocq M.A. (2020). Neurodevelopmental disorders—The history and future of a diagnostic concept. Dialogues Clin. Neurosci..

[60] Mullin A.P., Gokhale A., Moreno-De-Luca A., Sanyal S., Waddington J.L., Faundez V. (2013). Neurodevelopmental disorders: Mechanisms and boundary definitions from genomes, interactomes and proteomes. Transl. Psychiatry.

[61] Bonti E., Zerva I.K., Koundourou C., Sofologi M. (2024). The High Rates of Comorbidity among Neurodevelopmental Disorders: Reconsidering the Clinical Utility of Distinct Diagnostic Categories. J. Pers. Med..

[62] Bertollo A.G., Puntel C.F., da Silva B.V., Martins M.L., Bagatini M.D., Ign’acio Z.M. (2025). Neurobiological Relationships Between Neurodevelopmental Disorders and Mood Disorders. Brain Sci..

[63] Owen M.J., O’Donovan M.C. (2017). Schizophrenia and the neurodevelopmental continuum: Evidence from genomics. World Psychiatry.

[64] Stoccoro A., Conti E., Scaffei E., Calderoni S., Coppedè F., Migliore L., Battini R. (2023). DNA Methylation Biomarkers for Young Children with Idiopathic Autism Spectrum Disorder: A Systematic Review. Int. J. Mol. Sci..

[65] Hamza M., Halayem S., Bourgou S., Daoud M., Charfi F., Belhadj A. (2019). Epigenetics and ADHD: Toward an Integrative Approach of the Disorder Pathogenesis. J. Atten. Disord..

[66] Mirkovic B., Chagraoui A., Gerardin P., Cohen D. (2020). Epigenetics and Attention-Deficit/Hyperactivity Disorder: New Perspectives?. Front. Psychiatry.

[67] Fu G.H., Chen W., Li H.M., Wang Y.F., Liu L., Qian Q.J. (2021). A potential association of RNF219-AS1 with ADHD: Evidence from categorical analysis of clinical phenotypes and from quantitative exploration of executive function and white matter microstructure endophenotypes. CNS Neurosci. Ther..

[68] Zhang S.F., Gao J., Liu C.M. (2019). The Role of Non-Coding RNAs in Neurodevelopmental Disorders. Front. Genet..

[69] Liaci C., Prandi L., Pavinato L., Brusco A., Maldotti M., Molineris I., Oliviero S., Merlo G.R. (2022). The Emerging Roles of Long Non-Coding RNAs in Intellectual Disability and Related Neurodevelopmental Disorders. Int. J. Mol. Sci..

[70] Smigielski L., Jagannath V., Rössler W., Walitza S., Grünblatt E. (2020). Epigenetic mechanisms in schizophrenia and other psychotic disorders: A systematic review of empirical human findings. Mol. Psychiatry.

[71] Lordan R., Storni C., De Benedictis C.A., Grabrucker A.M. (2021). Autism Spectrum Disorders: Diagnosis and Treatment. Autism Spectrum Disorders.

[72] Márquez-Caraveo M.E., Rodríguez-Valentín R., Pérez-Barrón V., Vázquez-Salas R.A., Sánchez-Ferrer J.C., De Castro F., Allen-Leigh B., Lazcano-Ponce E. (2021). Children and adolescents with neurodevelopmental disorders show cognitive heterogeneity and require a person-centered approach. Sci. Rep..

[73] Aldinger K.A., Lane C.J., Veenstra-Van der Weele J., Levitt P. (2015). Patterns of Risk for Multiple Co-Occurring Medical Conditions Replicate Across Distinct Cohorts of Children with Autism Spectrum Disorder. Autism Res..

[74] Patel S., Han V.X., Keating B.A., Nishida H., Mohammad S., Jones H., Dale R.C. (2025). NDDECHO: A standardised digital assessment tool to capture early life environmental and inflammatory factors for children with neurodevelopmental disorders. Brain Behav. Immun. Health.

[75] Grandjean P., Landrigan P.J. (2006). Developmental neurotoxicity of industrial chemicals. Lancet.

[76] Grandjean P., Landrigan P.J. (2014). Neurobehavioural effects of developmental toxicity. Lancet Neurol..

[77] Frances L., Quintero J., Fernández A., Ruiz A., Caules J., Fillon G., Hervás A., Soler C.V. (2022). Current state of knowledge on the prevalence of neurodevelopmental disorders in childhood according to the DSM-5: A systematic review in accordance with the PRISMA criteria. Child. Adolesc. Psychiatry Ment. Health.

[78] Bougeard C., Picarel-Blanchot F., Schmid R., Campbell R., Buitelaar J. (2024). Prevalence of Autism Spectrum Disorder and Co-Morbidities in Children and Adolescents: A Systematic Literature Review. Focus.

[79] Baio J., Wiggins L., Christensen D.L., Maenner M.J., Daniels J., Warren Z., Kurzius-Spencer M., Zahorodny W., Robinson C.R., White T. (2018). Prevalence of Autism Spectrum Disorder Among Children Aged 8 Years—Autism and Developmental Disabilities Monitoring Network, 11 Sites, United States, 2014. MMWR Surveill. Summ..

[80] O’Sharkey K., Mitra S., Paik S.-A., Chow T., Cockburn M., Ritz B. (2025). Trends in the Prevalence of Autism Spectrum Disorder in California: Disparities by Sociodemographic Factors and Region Between 1990–2018. J. Autism Dev. Disord..

[81] Fombonne E., MacFarlane H., Salem A.C. (2021). Epidemiological surveys of ASD: Advances and remaining challenges. J. Autism Dev. Disord..

[82] Sacco R., Camilleri N., Eberhardt J., Umla-Runge K., Newbury-Birch D. (2023). The Prevalence of Autism Spectrum Disorder in Europe. Autism Spectrum Disorders—Recent Advances and New Perspectives.

[83] Issac A., Halemani K., Shetty A., Thimmappa L., Vijay V., Koni K., Mishra P., Kapoor V. (2025). The global prevalence of autism spectrum disorder in children: A systematic review and meta-analysis. Osong Public Health Res. Perspect..

[84] Lahiri D.K., Maloney B., Riyaz B.M., Ge Y.W., Zawia N.H. (2007). How and when environmental agents and dietary factors affect the course of Alzheimer’s disease: The “LEARn” model (Latent Early-Life Associated Regulation) may explain the triggering of AD. Curr. Alzheimer Res..

[85] Burgio E. (2015). Environment and Fetal Programming: The origins of some current ‘pandemics’. J. Pediatr. Neonatal Individ. Med..

[86] Lacagnina S. (2019). The Developmental Origins of Health and Disease (DOHaD). Am. J. Lifestyle Med..

[87] Raff R.A., Arthur W., Carroll S.B., Coates M.I., Wray G. (1999). Chronicling the birth of a discipline. Evol. Dev..

[88] Miranda-Dominguez O., Feczko E., Grayson D.S., Walum H., Nigg J.T., Fair D.A. (2018). Heritability of the human connectome: A connectotyping study. Netw. Neurosci..

[89] Bailo P., Piccinini A., Barbara G., Caruso P., Bollati V., Gaudi S. (2024). Epigenetics of violence against women: A systematic review of the literature. Environ. Epigenet..

[90] Carannante A., Giustini M., Rota F., Bailo P., Piccinini A., Izzo G., Bollati V., Gaudi S. (2025). Intimate partner violence and stress-related disorders: From epigenomics to resilience. Front. Glob. Women’s Health.

[91] Tuulari J.J., Bourgery M., Iversen J., Koefoed T.G., Ahonen A., Ahmedani A., Kataja E.-L., Karlsson L., Barrès R., Karlsson H. (2025). Exposure to childhood maltreatment is associated with specific epigenetic patterns in sperm. Mol. Psychiatry.

[92] Stenz L., Schechter D.S., Serpa S.R., Paoloni-Giacobino A. (2018). Intergenerational Transmission of DNA Methylation Signatures Associated with Early Life Stress. Curr. Genom..

[93] An E., Delgadillo D.R., Yang J., Agarwal R., Labus J.S., Pawar S., Leitman M., Kilpatrick L.A., Bhatt R.R., Vora P. (2024). Stress-resilience impacts psychological wellbeing as evidenced by brain–gut microbiome interactions. Nat. Ment. Health.

[94] Roth T.L., Sweatt J.D. (2011). Annual Research Review: Epigenetic mechanisms and environmental shaping of the brain during sensitive periods of development. J. Child Psychol. Psychiatry.

[95] Tran N.Q.V., Miyake K. (2017). Neurodevelopmental Disorders and Environmental Toxicants: Epigenetics as an Underlying Mechanism. Int. J. Genom..

[96] Bose R., Spulber S., Ceccatelli S. (2023). The threat posed by environmental contaminants on neurodevelopment: What can we learn from neural stem cells?. Int. J. Mol. Sci..

[97] Antoniou E.E., Otter R. (2024). Phthalate exposure and neurotoxicity in children: A systematic review and meta-analysis. Int. J. Public Health.

[98] Sun C., Huang C., Yu C.W. (2023). Environmental exposure and infants’ health. Indoor Built Environ..

[99] FIsmail Y., Fatemi A., Johnston M.V. (2017). Cerebral plasticity: Windows of opportunity in the developing brain. Eur. J. Paediatr. Neurol..

[100] Lanphear B.P., Hornung R., Khoury J., Yolton K., Baghurst P., Bellinger D.C., Canfield R.L., Dietrich K.N., Bornschein R., Greene T. (2005). Low-level environmental lead exposure and children’s intellectual function: An international pooled analysis. Environ. Health Perspect..

[101] Kumar A., Kumar A., Cabral-Pinto M.M.S., Chaturvedi A.K., Shabnam A.A., Subrahmanyam G., Mondal R., Gupta D.K., Malyan S.K., Kumar S.S. (2020). Lead Toxicity: Health Hazards, Influence on Food Chain, and Sustainable Remediation Approaches. Int. J. Environ. Res. Public Health.

[102] Johansson C., Castoldi A.F., Onishchenko N., Manzo L., Vahter M., Ceccatelli S. (2007). Neurobehavioural and molecular changes induced by methylmercury exposure during development. Neurotox. Res..

[103] Farıas P., Hern’andez-Bonilla D., Moreno-Mac’ıas H., Montes-L’opez S., Schnaas L., Texcalac-Sangrador J.L., R’ıos C., Riojas-Rodrıguez H. (2022). Prenatal Co-Exposure to Manganese, Mercury, and Lead, and Neurodevelopment in Children during the First Year of Life. Int. J. Environ. Res. Public Health.

[104] Appleton A.A., Jackson B.P., Karagas M., Marsit C.J. (2017). Prenatal exposure to neurotoxic metals is associated with increased placental glucocorticoid receptor DNA methylation. Epigenetics.

[105] Perera F., Miao Y., Ross Z., Rauh V., Margolis A., Hoepner L., Riley K.W., Herbstman J., Wang S. (2024). Prenatal exposure to air pollution during the early and middle stages of pregnancy is associated with adverse neurodevelopmental outcomes at ages 1 to 3 years. Environ. Health.

[106] Grova N., Schroeder H., Olivier J.L., Turner J.D. (2019). Epigenetic and Neurological Impairments Associated with Early Life Exposure to Persistent Organic Pollutants. Int. J. Genom..

[107] Botnaru A.A., Lupu A., Morariu P.C., Jităreanu A., Nedelcu A.H., Morariu B.A., Anton E., Di Gioia M.L., Lupu V.V., Dragostin O.M. (2025). Neurotoxic Effects of Pesticides: Implications for Neurodegenerative and Neurobehavioral Disorders. J. Xenobiot..

[108] Brannen K.C., Devaud L.L.B., Liu J.C., Lauder J.M. (1998). Prenatal exposure to neurotoxicants dieldrin or lindane alters tert-Butylbicyclophosphorothionate binding to GABA_A_ receptors in fetal rat brainstem. Dev. Neurosci..

[109] Van Melis L.V.J., Bak T., Peerdeman A.M., van Kleef R.G.D.M., Wopken J.P., Westerink R.H.S. (2025). Acute, prolonged, and chronic exposure to organochlorine insecticides evoke differential effects on in vitro neuronal activity and network development. Neurotoxicology.

[110] Rauh V.A., Garfinkel R., Perera F.P., Andrews H.F., Hoepner L., Barr D.B., Whitehead R., Tang D., Whyatt R.W. (2006). Impact of prenatal chlorpyrifos exposure on neurodevelopment in the first 3 years of life among inner-city children. Pediatrics.

[111] Roberts J.R., Dawley E.H., Reigart J.R. (2019). Children’s low-level pesticide exposure and associations with autism and ADHD: A review. Pediatr. Res..

[112] Bertoletti A.C.C., Peres K.K., Faccioli L.S., Vacci M.C., Mata I.R.D., Kuyven C.J., Bosco S.M.D. (2022). Early exposure to agricultural pesticides and the occurrence of autism spectrum disorder: A systematic review. Rev. Paul. Pediatr..

[113] Atladottir H.O., Thorsen P., Ostergaard L., Schendel D.E., Lemcke S., Abdallah M., Parner E.T. (2010). Maternal infection requiring hospitalization during pregnancy and autism spectrum disorders. J. Autism Dev. Disord..

[114] Zerbo O., Iosif A.-M., Walker C., Ozonoff S., Hansen R.L., Hertz-Picciotto I. (2013). Is maternal influenza or fever during pregnancy associated with autism or developmental delays? Results from the CHARGE (Childhood Autism Risks from Genetics and Environment) study. J. Autism Dev. Disord..

[115] Kwon H.K., Choi G.B., Huh J.R. (2022). Maternal inflammation and its ramifications on fetal neurodevelopment. Trends Immunol..

[116] Ayoub G. (2025). Neurodevelopmental impact of maternal immune activation and autoimmune disorders, environmental toxicants and folate metabolism on autism spectrum disorder. Curr. Issues Mol. Biol..

[117] Keil A., Daniels J.L., Forssen U., Hultman C., Cnattingius S., Soderberg K.C., Feychting M., Sparen P. (2010). Parental autoimmune diseases associated with autism spectrum disorders in offspring. Epidemiology.

[118] Atladottir H.O., Pedersen M.G., Thorsen P., Mortensen P.B., Deleuran B., Eaton W.W., Parner E.T. (2009). Association of family history of autoimmune diseases and autism spectrum disorders. Pediatrics.

[119] MSpann N., Timonen-Soivio L., Suominen A., Cheslack-Postava K., McKeague I.W., Sourander A., Brown A.S. (2019). Proband and familial autoimmune diseases are associated with proband diagnosis of autism spectrum disorders. J. Am. Acad. Child Adolesc. Psychiatry.

[120] Labouesse M.A., Dong E., Grayson D.R., Guidotti A., Meyer U. (2015). Maternal immune activation induces *GAD1* and *GAD2* promoter remodeling in the offspring prefrontal cortex. Epigenetics.

[121] Dudova I., Horackova K., Hrdlicka M., Balastik M. (2020). Can maternal autoantibodies play an etiological role in ASD development?. Neuropsychiatr. Dis. Treat..

[122] Braunschweig D., Krakowiak P., Duncanson P., Boyce R., Hansen R.L., Ashwood P., Hertz-Picciotto I., Pessah I.N., Van de Water J. (2013). Autism-specific maternal autoantibodies recognize critical proteins in developing brain. Transl. Psychiatry.

[123] Jones K.L., Van de Water J. (2019). Maternal autoantibody related autism: Mechanisms and path-ways. Mol. Psychiatry.

[124] Ramirez-Celis A., Croen L.A., Yoshida C.K., Alexeeff S.E., Schauer J., Yolken R.H., Ashwood P., Van de Water J. (2022). Maternal autoantibody profiles as biomarkers for ASD and ASD with co-occurring intellectual disability. Mol. Psychiatry.

[125] Bruce M.R., Couch A.C.M., Grant S., McLellan J., Ku K., Chang C., Bachman A., Matson M., Berman R.F., Maddock R.J. (2023). Altered behavior, brain structure, and neurometabolites in a rat model of autism-specific maternal autoantibody exposure. Mol. Psychiatry.

[126] Banik A., Kandilya D., Ramya S., Stünkel W., Chong Y.S., Dheen S.T. (2017). Maternal factors that induce epigenetic changes contribute to neurological disorders in offspring. Genes.

[127] Lussier A.A., Bodnar T.S., Weinberg J. (2021). Intersection of epigenetic and immune alterations: Implications for fetal alcohol spectrum disorder and mental health. Front. Neurosci..

[128] Smith A., Kaufman F., Sandy M.S., Cardenas A. (2020). Cannabis exposure during critical windows of development: Epigenetic and molecular pathways implicated in neuropsychiatric disease. Curr. Environ. Health Rep..

[129] Alvarez-Mejıa D., Rodas J.A., Leon-Rojas J.E. (2025). From womb to mind: Prenatal epigenetic influences on mental health disorders. Int. J. Mol. Sci..

[130] Baroutis D., Sotiropoulou I.M., Mantzioros R., Theodora M., Daskalakis G., Antsaklis P. (2025). Prenatal maternal stress and long-term neurodevelopmental outcomes: A narrative review. J. Perinat. Med..

[131] Yao B., Cheng Y., Wang Z., Li Y., Chen L., Huang L., Zhang W., Chen D., Wu H., Tang B. (2017). DNA N6-methyladenine is dynamically regulated in the mouse brain following environmental stress. Nat. Commun..

[132] Ohuma E., Moller A.-B., Bradley E., Chakwera S., Hussain-Alkhateeb L., Lewin A., Okwaraji Y.B., Mahanani W.R., Johansson E.W., Lavin T. (2023). National, regional, and global estimates of preterm birth in 2020, with trends from 2010: A systematic analysis. Lancet.

[133] Cervantes E.M., Girard S. (2025). Placental inflammation in preterm premature rupture of membranes and risk of neurodevelopmental disorders. Cells.

[134] Lyall K., Song L., Botteron K., Croen L.A., Dager S.R., Fallin M.D., Hazlett H.C., Kauffman E., Landa R., Ladd-Acosta R. (2020). The association between parental age and autism-related outcomes in children at high familial risk for autism. Autism Res..

[135] Krug A., Wöhr M., Seffer D., Rippberger H., Sungur A.Ö., Dietsche B., Stein F., Sivalingam S., Forstner A.J., Witt S.H. (2020). Advanced paternal age as a risk factor for neurodevelopmental disorders: A translational study. Mol. Autism.

[136] Weaver I., Cervoni N., Champagne F., D’Alessio A.C., Sharma S., Seckl J.R., Dymov S., Szyf M., Meaney M.J. (2004). Epigenetic programming by maternal behavior. Nat. Neurosci..

